# Fibronectin Fibers Progressively Lose Their Tension in Invasive Human Breast Carcinoma while Being Tensed in DCIS and Healthy Breast Tissue

**DOI:** 10.1002/advs.202404351

**Published:** 2025-06-05

**Authors:** Arnaud Miéville, Charlotte M. Fonta, Cornelia Leo, Lucine Christe, Jörg Goldhahn, Gad Singer, Viola Vogel

**Affiliations:** ^1^ Laboratory of Applied Mechanobiology Institute of Translational Medicine Department of Health Sciences and Technology ETH Zürich Zürich Switzerland; ^2^ Breast Center Department of Gynecology Kantonsspital Baden Baden Switzerland; ^3^ Institute of Tissue Medicine and Pathology University of Bern Bern Switzerland; ^4^ Institute of Translational Medicine Department of Health Sciences and Technology ETH Zürich Zürich Switzerland; ^5^ Institute of Pathology Kantonsspital Baden Baden Switzerland

**Keywords:** breast cancer, ductal carcinoma in situ (DCIS), extracellular matrix, mechanobiology, tensional state of Fibronectin

## Abstract

Extracellular matrix (ECM) remodeling plays critical roles in cancer progression and involves alterations in its composition and biophysical properties. Aggressiveness and malignancy of solid tumors are strongly correlated with tissue stiffening, mainly due to upregulated ECM production and cross‐linking. However, nothing is known about the tensional alterations that occur at the single‐fiber level during tumorigenesis in humans. The well‐validated peptide tension probe (FnBPA5) now reveals that Fibronectin fibers lose their tension as invasive tumors progress while they are stretched in healthy human breast tissue stroma and in ductal carcinoma in situ (DCIS), the non‐invasive precursor of breast cancer. In invasive carcinomas, cancer cells, cancer‐associated fibroblasts (CAFs) and infiltrating immune cells (cytotoxic T cells and regulatory T cells), are predominantly located in proximity to untensed Fibronectin fibers. This is significant, as Fibronectin fiber stretching can mechano‐regulate the reciprocal cell‐ECM crosstalk and the bioavailability of ECM‐bound molecules. Not only tissue stiffening, but also the accumulation of untensed Fibronectin fibers may serve as a mechanical biomarker that correlates with tumor grade. Loss of Fibronectin fiber tension may play a central role in regulating tumor invasiveness. This suggests that physically altered ECM fibers can be exploited for stroma‐targeted drug delivery and immunotherapy.

## Graphical abstract

Extracellular matrix remodeling is crucial in cancer progression. Using a peptide to probe the tension of ECM fibers, it was found that Fibronectin fibers gradually lose tension as human breast carcinoma progresses, while their tension is maintained in ductal carcinoma in situ (DCIS), the non‐invasive precursor of breast cancer. This loss of tension is associated with the presence of T cells, cancer‐associated fibroblasts(CAFs), and cancer cells, prone to be exploited for stroma‐targeted drug delivery and immunotherapy.

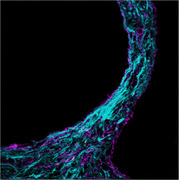

## 1 Introduction

Breast carcinomas are one of the most common cancers worldwide, accounting for ≈ 11% of new cancer cases and causing more than half a million deaths worldwide each year.^[^ ^1^ ^]^ Tumor growth and malignancy go hand‐in‐hand with extracellular matrix (ECM) remodeling, mostly driven by myofibroblastic cancer‐associated fibroblasts (CAFs).^[^ ^2^, ^3^, ^4^, ^5^, ^6^ ^]^ In numerous solid tumors, the severely altered ECM can constitute up to 60% of the tumor mass.^[^ ^6^ ^]^ The stiffened ECM meshwork not only facilitates tumor growth, but also acts as a barrier, hindering the migration of immune cells and impeding effective cancer therapy due to the high deposition and crosslinking of collagen fibers, among others.^[^ ^6^, ^7^, ^8^, ^9^ ^]^ Not only does the ECM composition change in tumor microenvironments, but its physical and mechanical properties are altered too, driven by the reciprocal interplay between the ECM and the cancer‐associated cells. This leads to interstitial hypertension due to hyperpermeable blood vessels, increased solid stress, increased viscoelasticity, and tissue stiffness, as driven by the enhanced contractility of activated fibroblasts, as well as to increased matrix deposition, fiber alignment and ECM cross‐linking.^[^ ^9^, ^10^, ^11^, ^12^, ^13^, ^14^, ^15^, ^16^, ^17^, ^18^ ^]^ Cancer‐associated cells progressively adapt their phenotypes to this altered biophysical microenvironment, further enhancing the tumor's malignancy.^[^ ^9^, ^19^ ^]^ Recent efforts to understand and exploit the “physics of cancer” have led to the emergence of diagnostic methods based on probing physical features of the stroma, and using these physical traits as therapeutic targets to interfere with cancer development and progression.^[^ ^10^, ^11^, ^20^, ^21^, ^22^, ^23^, ^24^, ^25^, ^26^ ^]^ While the primary focus has been on quantifying changes in the stiffness of the tumor microenvironment and in the collagen fiber deposition,^[^ ^9^, ^24^, ^27^, ^28^, ^29^, ^30^ ^]^ cells locally exert mechanical forces on individual ECM fibers, altering their arrangement and tension. Not only does this change the Young's modulus of individual ECM fibers,^[^ ^31^ ^]^ but fiber stretching can also modulate the presentation of molecular binding sites, either by exposing cryptic binding sites, or by destroying other binding site motifs.^[^ ^31^, ^32^, ^33^, ^34^, ^35^, ^36^ ^]^ ECM proteins can thus serve as mechano‐chemical signaling converters and thereby contributing to cellular mechanotransduction. This is particularly true for Fibronectin, one of the most abundant proteins distributed ubiquitously in the ECM, that plays a crucial role in cell‐ECM interactions and as driver of tumor growth and metastasis.^[^ ^3^, ^37^, ^38^, ^39^ ^]^ The affinity of many of its binding partners is regulated by Fibronectin's fiber tension, including the closed conformation of Tissue transglutaminase 2 (TG2),^[^ ^35^ ^]^ as well as interleukin‐7 (IL‐7),^[^ ^40^ ^]^ and integrin α_V_β_3_ versus α_5_β_1_.^[33,^ ^41^ ^]^ TG2 and IL‐7 are major players in cancer stroma remodeling and tune immune cell responses,^[^ ^42^, ^43^ ^]^ while integrin α_V_β_3_ and α_5_β_1_ are both known to steer tumor progression by promoting cell adhesion, migration, and metastasis.^[^ ^15^, ^44^ ^]^

Mammographically dense breast tissue is one of the major risk factors for the development of breast cancer.^[^ ^14^, ^45^, ^46^, ^47^ ^]^ Invasive tumors are characterized by a stiff ECM,^[^ ^10^, ^11^, ^47^ ^]^ rich in collagen type I fibers,^[^ ^27^, ^47^, ^48^ ^]^ whose increased fiber alignment in tumors^[^ ^27^, ^49^ ^]^ positively correlates with increased malignancy^[^ ^15^, ^50^ ^]^ and aggressiveness.^[^ ^24^, ^48^ ^]^ Information on how the tensional state of individual ECM fibers might be altered in cancer and how this might impact tumorigenesis has been lacking, until recently, due to the lack of tools to map ECM fiber tension at the molecular level in organs. To address this challenge, we introduced FnBPA5, a mechano‐regulated Fibronectin fiber tension probe that binds to the N‐terminal Fibronectin type I modules FnI_2‐5_.^[^ ^34^, ^51^, ^52^ ^]^ This peptide is derived from a bacterial adhesin that has been optimized for millions of years to help bacteria bind to cleaved Fibronectin fibers of wound sites.^[^ ^34^ ^]^ This peptide recognizes Fibronectin's N‐terminal equilibrium structure with nanomolar affinity, yet its affinity can be mechanically downregulated, as fiber stretching can destroy its multivalent binding motif.^[^ ^34^, ^52^ ^]^ While each of the N‐terminal Fibronectin Type I domains contains intradomain disulfide bonds stabilizing their cores, steered molecular dynamics (SMD) revealed that tensile forces stretch out the intermittent residues linking the type I domains, thereby extending the length of the FnI_1‐5_ fragment by more than 30% resulting in a structural mismatch.^[^ ^52^ ^]^ Fibronectin fiber stretch assays confirmed that FnBPA5 binds with nm affinity to untensed Fibronectin fibers, while fiber stretching gradually reduces its affinity.^[^ ^51^ ^]^ Labeling native Fibronectin fibers in cell culture with two different strain probes, i.e., a Fibronectin‐FRET probe^[^ ^53^, ^54^ ^]^ versus the FnBPA5 probe revealed that these two complementary read‐outs correlated very well over a large range of Fibronectin fiber strains.^[^ ^51^, ^55^ ^]^ This was an important validation of the tension probe, as these two probes exploit orthogonal physical mechanisms. In contrast, the use of a Fibronectin polyclonal antibody allows us to stain for any type and physical state of Fibronectin fibers.^[^ ^34^, ^51^, ^52^, ^56^ ^]^ This enabled us for the first time, to map the tensional state of Fibronectin fibers in tissue cryosections from healthy mice organs and induced cancer models.^[^ ^51^, ^56^, ^57^ ^]^ In these mouse tumor models, we previously discovered unexpectedly that large fractions of Fibronectin fibers are untensed. This is in stark contrast to healthy organs, where Fibronectin fibers were found to be mostly kept under high tension.^[^ ^56^ ^]^ A spatial correlation was also observed between untensed Fibronectin fibers and aligned dense collagen fiber bundles as visualized by Second Harmonic Generation (SHG), and surprisingly, they are both present in proximity to the highly contractile α‐smooth muscle actin (α‐SMA) positive myofibroblastic CAFs.^[^ ^51^, ^56^ ^]^ These regions, rich in untensed Fibronectin fibers, also correlate with the presence of tenascin C (TNC), a well‐known cancer biomarker.^[^ ^57^, ^58^ ^]^ Colocalization was also seen in tumor matrix tracks in mouse models, which were further enriched in leukocytes, and recent work suggests that these structures have immunosuppressive capabilities.^[^ ^57^, ^59^ ^]^ These murine studies highlighted the need not only for a fundamental understanding of the processes of ECM remodeling in tumorigenesis, but also in particular, the poorly understood role of untensed Fibronectin fibers in cancer stroma, potentially steering tumor growth and tumor immunogenicity.^[^ ^51^, ^56^, ^57^ ^]^

The predictive power of murine models is under debate due to differences in tumor morphology and traits as tumors in mice are typically seeded in young mice and rapidly grow within a few weeks. We thus asked here whether untensed Fibronectin fibers are also seen in human breast cancer tissues and confirmed significant alterations in the tensional state of Fibronectin fibers in clinically classified human breast carcinomas. Using ductal carcinoma in situ (DCIS), and invasive carcinomas of different grades, we asked here how the distribution of Fibronectin fiber tensions changes during tumor progression and how this correlates with tumor microenvironment remodeling. We further quantified the spatial proximity of untensed Fibronectin fibers to α−SMA expressing CAFs, as well as cancer cells and immune cells, as tension alterations can switch the affinity for many of Fibronectin's binding partners. We characterized the ECM‐fiber‐modulated mechanobiological landscape in invasive human breast carcinomas, DCIS, and healthy breast tissues. Our findings not only enhance our fundamental understanding of the reciprocal interactions between cells and their ECM, but also, through cross‐validation, highlight novel mechano‐regulated ECM biomarkers that could be utilized for the development of tumor stroma‐targeted therapies.

## 2 Results

Tissue cryosections from human breast carcinomas, i.e., from non‐invasive (DCIS) and from invasive carcinomas, as well as from healthy breast tissues harvested from either breast reduction surgery or from healthy tissue adjacent to tumors were obtained from the Kantonsspital Baden (KSB) (Baden, Switzerland) and from the Tissue Bank Bern (TBB) (Bern, Switzerland). At the time of tissue donation, the patients had not yet undergone chemotherapy or radiotherapy. Note that staining with the Cy5‐FnBPA5 tension probe is not compatible with formalin‐fixed, paraffin‐embedded (FFPE) tissue sections, routinely used in pathology for resected tumors, due to partial protein denaturation during the embedding protocol. Cy5‐FnBPA5 can only be used to stain tissue cryosections, thereby limiting access to patient tumor samples. Because of this limitation, and because of the high molecular heterogeneity of tumors, even within the same subtypes, the routinely conducted clinical classifications were used here, distinguishing tumors by size and grade (**Table** **1**). A complete pathological diagnosis of each patient, performed by clinical pathologists from KSB and TBB, is provided in Table S1 (Supporting Information). Each patient sample used in this study was assigned a patient‐specific color code to interconnect the images and data quantifications shown in different Figures and Tables.

**Table 1:** Overview of breast tissue samples from patients and their characteristic features. In the clinical classification performed by pathologists, the “p” stands for “pathological stage”, also called surgical stage, identified as tumors from a biopsy.^[^ ^60^ ^]^ The “T” followed by a number between 0 and 4 refers to the size of the tumor, with increasing values associated with larger tumor sizes. T1 (a to c) refers to tumors up to 2 cm large. Within the T1 category, tumors are further classed into sub‐categories according to their size, with the letters a, b, and c for tumor sizes in the ranges [0.1 cm and 0.5 cm], [0.5 cm to 1 cm], and [1 cm to 2 cm] respectively. The size of tumors smaller than 0.1 cm is referred to as “Tmi”. T2 refers to tumors between 2 cm and 5 cm, and T3 to tumors larger than 5 cm. Finally, Tis refers to carcinoma in situ (DCIS) or Paget disease, the non‐invasive precursor of breast carcinoma. A complete list can be found in Table S1 (Supporting Information). Each patient was attributed with a specific color as shown in Table S1 (Supporting Information) too.

	Number of Samples	Average age of female patient	Invasive growth	Morphology of collagen fiber bundles visualized by SHG	Cy5‐FnBPA5 bound to untensed Fibronectin fibers	TNC locations
**Healthy**	*n* = 10	65	‐	Wavy	Very low, almost absent/undetectable	Rare/no TNC detected
**DCIS (Tis)**	*n* = 4	53	No	Wavy, some alignment	Very low, almost absent	Very little TNC detected
**Invasive** **pT1c**	*n* = 5	68	Yes	Dense aligned fiber bundles	High, fiber‐like	TNC along the untensed Fibronectin fibers
**Invasive** **pT2**	*n* = 11	60	Yes	Dense aligned fiber bundles	High, fiber‐like	TNC along the untensed Fibronectin fibers
**Invasive** **pT3**	*n* = 1	73	Yes	Dense aligned fiber bundles	High, fiber‐like	TNC along the untensed Fibronectin fibers

### 2.1 Invasive Human Breast Tumors Are Enriched in Untensed Fibronectin Fibers, Which Are Absent in Healthy Breast Tissues

**Figure 1:**
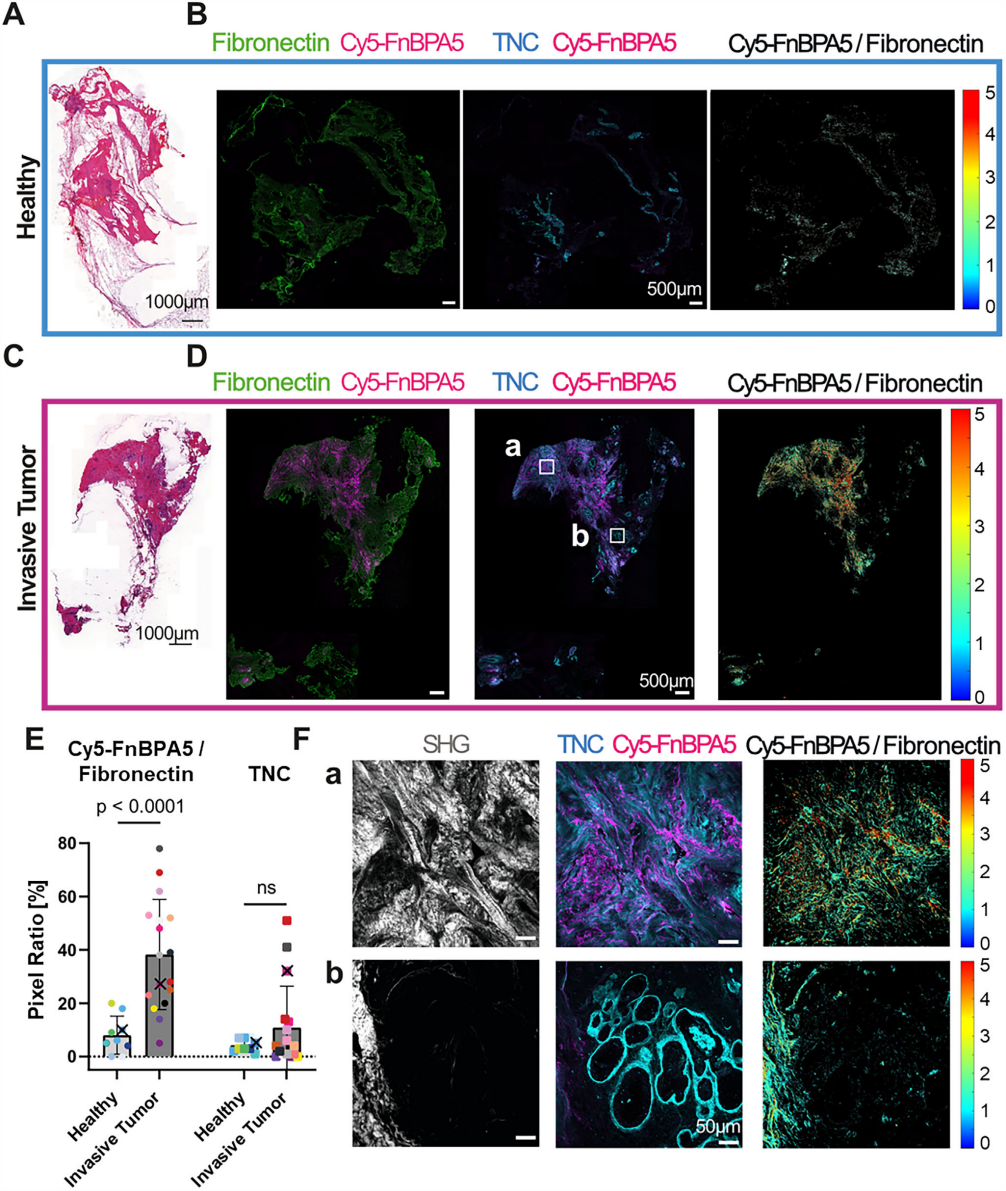
Invasive human breast carcinoma cryosections show enhanced Cy5‐FnBPA5 peptide binding to untensed Fibronectin fibers, while its binding is significantly lower in human healthy breast cryosections. A,B) Human healthy breast cryosections stained with H&E (A), together with confocal overview images from tissues stained with a polyclonal Fibronectin antibody (green) to visualize all Fibronectin fibers, and with the Cy5‐FnBPA5 tension probe (magenta) to visualize untensed Fibronectin fibers, as well as with a monoclonal TNC antibody (cyan) (B). The H&E stained tissue sections were adjacent to the immunostained slices in the original tissues. C,D) Invasive breast carcinoma cryosections stained with H&E (C), or with a Fibronectin polyclonal antibody (green), a TNC monoclonal antibody (cyan), and with the Cy5‐FnBPA5 tension probe (magenta) (D). TNC and Cy5‐FnBPA5 are both detected in tumors (D), but at significantly lower levels in healthy breast cryosections (B). All images are framed with the patient color and originate from patients #1, #20, respectively (Table S1, Supporting Information). Scale bars full H&E tissues: 1000 µm; scale bars full IF tissues: 500 µm. The Cy5‐FnBPA5/Fibronectin ratios provide pixel‐by‐pixel intensity ratios. These ratios are relative (a.u.), as the use of two different fluorophores does not allow to quantify their absolute molecular ratios. E) Pixel ratios for Cy5‐FnBPA5 give the percentage of positive pixels above a defined threshold for the Cy5‐FnBPA5 channel (circle), normalized to the total number of positive pixels for the Fibronectin polyclonal antibody. TNC signal was assessed as the percentage of positive pixels above a defined threshold for the TNC channels (square), normalized to the total number of pixels in the studied area. Several cryosections were analyzed for each patient and the average of their respective pixel ratios was plotted. To link the confocal images with the representative Patient specific data point, the corresponding dots were marked with a cross. Each colored symbol represents one patient (Tumor: *n* = 17, Healthy: *n* = 10), with the associated patient information as provided in Table S1 (Supporting Information). Mean ±SD. Mann‐Whitney test. F) Higher resolution zoom‐ins of the tumor cryosection reveal the high heterogeneity within the tumor tissue from the same patient, with some areas highly fibrotic, presenting an enhanced untensed Fibronectin fiber content, strong TNC counts, and aligned collagen fiber bundles detected with SHG (F.a), while others were non‐fibrotic, showing no fiber‐like TNC and untensed Fibronectin fibers, and significantly lower SHG intensities (F.b). Scale bars for zoom‐in areas: 50 µm.

Stitched overview images of H&E stains for two representative patients, as well as the corresponding fluorescence images from adjacent cryosections are shown in **Figure** **1**, illustrating the tissue heterogeneities as expected, while a collection of representative confocal zoom‐in images for each of the patients can be found in Figure S1 (Supporting Information). Cryosections of healthy tissues (Figure 1A,B) and invasive tumors (Figure 1C,D) show that the ECM of both healthy and tumor cryosections is rich in Fibronectin fibers, as expected. The intense pink signals in both of the H&E stained tissues reveal the presence of cytoplasmic proteins and ECM fibers (Figure 1A,C). The more intense darker‐pink signals in the tumor cryosections indicate the high collagen fiber content and the increased number of cell nuclei, which reflect the increased cellularity of tumor tissues (Figure 1C, Supporting Information). While healthy and the cancerous breast tissues show abundant Fibronectin (pAB, green), almost no untensed Fibronectin fibers (Cy5‐FnBPA5 binding, magenta) are detected in the healthy tissues (Figure 1B, Supporting Information). In contrast, significantly enhanced Cy5‐FnBPA5 binding is seen in invasive tumors showing heterogeneous patches of fiber relaxation with areas highly stretched and some highly untensed (Figure 1D). Locations of Cy5‐FnBPA5 binding correlate with strong local TNC staining (Figure 1D). TNC, whose presence is highly synonym of fibrotic environments,^[^ ^58^, ^61^ ^]^ is abundantly expressed in tumors (Figure 1D), while sparse in healthy tissues (Figure 1B). These observations correlate with the fractional pixel ratio calculated for Cy5‐FnBPA5 signals (Figure 1E) upon analysis of all patient tissues. The pixel ratio is defined as the proportion of positive pixels above a defined threshold for each stain relative to the total number of pixels in the examined area. For Cy5‐FnBPA5, a ratiometric analysis was performed by normalizing the number of Cy5‐FnBPA5 positive pixels to the total number of positive pixels for the Fibronectin polyclonal antibody. When comparing tumor and healthy tissue cryosections, ≈40% of pixels are positive for Cy5‐FnBPA5 in tumor tissues, while only 8% are found in healthy cryosections (Figure 1E). A similar trend is observed for the TNC pixel ratio, with ≈10% of the tumor tissues positive, while only ≈5% of TNC's positive pixels are found in healthy cryosections.

The tumor cryosection zoom‐in images of two different areas clearly depict the structural and ECM heterogeneities in a single tumor, as expected (Figure 1F). One area is rich in TNC and Cy5‐FnBPA5‐positive, thus in untensed Fibronectin fibers, colocalizing with a strong SHG signal originating from aligned dense collagen fiber bundles (Figure 1Fa). Whereas in another area, only TNC is present, with no Cy5‐FnBPA5 binding detected, nor much SHG (Figure 1Fb). Here, TNC is associated with circular patterns, as if lining duct structures, similar to those previously observed in healthy human breast tissues^[^ ^48^ ^]^ and in mammary carcinomas.^[^ ^62^ ^]^

### 2.2 DCIS Retained the Tensional Fibronectin Fiber Signature of Healthy Tissues, in Contrast to Invasive Breast Carcinoma

**Figure 2:**
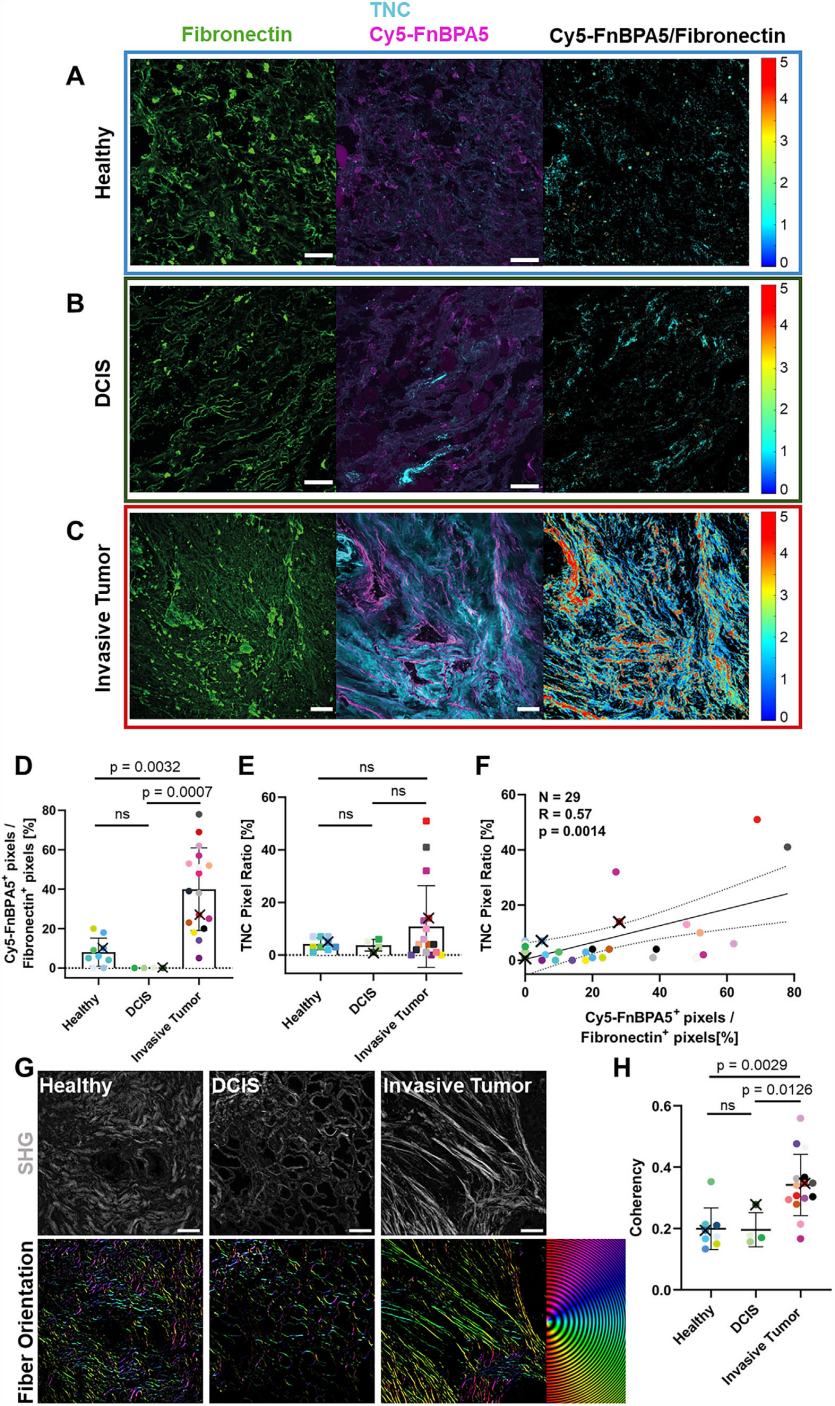
Extracellular matrix (ECM) and tensional Fibronectin fiber signatures for healthy breast tissues, as well as for non‐invasive (DCIS) and invasive breast carcinomas. A–C) Representative confocal images of cryosections stained with a polyclonal Fibronectin antibody (green) to visualize the presence of all Fibronectin fibers, co‐stained with Cy5‐FnBPA5 tension probe (magenta) to visualize the untensed Fibronectin fibers, and a monoclonal TNC antibody (cyan) for the following tissues: A) Healthy breast tissue, B) DCIS, and C) Invasive Tumor. The pixel‐by‐pixel Cy5‐FnBPA5/Fibronectin intensity ratios (a.u., not molecular ratio). All images are framed with the patient color and originate from patients #1, #11, #21, respectively (Table S1, Supporting Information). Scale bars: 50 µm. D) Pixel ratio for Cy5‐FnBPA5 gives the percentage of positive pixels above a defined threshold for the Cy5‐FnBPA5 channel, normalized to the total number of positive pixels for the Fibronectin polyclonal antibody. E) TNC pixel ratio was assessed as the percentage of positive pixels above a defined threshold for the TNC channel, normalized to the total number of pixels in the studied area. Several cryosections were analyzed for each patient and the average of their respective pixel ratios were plotted. Mean ± SD. Kruskal‐Wallis test with Dunn's multiple comparison test. Each dot represents one patient (Healthy: *n* = 10, DCIS: *n* = 4, Invasive Tumor: *n* = 17). F) Correlation plot between TNC and Cy5‐FnBPA5 signals. N = 29 patients, R = 0.57. Correlation is significant at the 0.01 level. To link the confocal images with the representative patient specific data point, the corresponding dots were marked with a cross in each graph. A compilation of images for the different patients is shown in Figure S1 (Supporting Information). G) SHG detection to localize dense bundles of collagen fibers (gray) for each condition (Healthy, DCIS, Invasive Tumor) together with their color‐coded orientations. H) Coherency analysis of collagen fibers (SHG). Several cryosections were analyzed for each patient, and the average of their coherency was plotted. Mean ± SD. Kruskal‐Wallis test with Dunn's multiple comparisons test. Each dot represents one patient (Healthy: *n* = 10, DCIS: *n* = 4, Invasive Tumor: *n* = 14).

To investigate how Fibronectin fiber tension might differ in invasive carcinomas versus non‐invasive ductal carcinomas in situ (DCIS), several confocal images were acquired for each sample (**Figure** **2**; Figure S1, Supporting Information). Similar to the observations made in the previous Figure (Figure 1B), healthy breast tissues, while rich in Fibronectin fibers, do not display a significant TNC signal, nor do they exhibit major Cy5‐FnBPA5 binding (Figure 2A; Figure S1, Supporting Information). The SHG signal shows a wavy collagen fiber pattern, as previously described in healthy breast tissues^[^ ^48^ ^]^ (Figure 2G, “SHG”). DCIS tissues present an ECM with a rather low fibrotic signature, with a comparatively low TNC signal, and a predominance of stretched Fibronectin fibers, as suggested by the high Fibronectin fiber content accompanied by low Cy5‐FnBPA5 binding (Figure 2B). No significant statistical differences are seen in the Cy5‐FnBPA5 binding between healthy and DCIS tissues. Confocal images of invasive tumors display strong TNC signals, as expected, and importantly these loci are associated with enhanced Cy5‐FnBPA5 binding and dense and aligned bundles of collagen fibers (Figure 2C,G; Figure S1, Supporting Information). Ratiometric pixel‐by‐pixel analysis of Cy5‐FnBPA5/Fibronectin intensities reveals major spatial heterogeneities in Fibronectin fiber relaxation within the invasive tumor stroma, with regions of both low and high fiber tension (Figure 2C). Note, however, that the Cy5‐FnBPA5 to total Fibronectin intensity ratios are relative (a.u.), as the use of two different fluorophores does not allow to quantify their absolute molecular ratios.

A significantly increased percentage of Cy5‐FnBPA5^+^ pixels is observed in invasive tumors compared to DCIS and healthy tissues, as revealed by quantitative pixel‐by‐pixel analysis of all images for each patient (Figure 2D), confirming the observations made in confocal images (Figure 2A–C; Figure S1, Supporting Information). While the total Fibronectin pixel ratio for each condition shows no significant differences (Figure S2, Supporting Information), the fraction of untensed Fibronectin fibers significantly increases in invasive carcinoma. Remarkably, a positive Pearson correlation was found between the presence of Cy5‐FnBPA5^+^ and TNC^+^ pixels across all tissues [*r* = 0.57, *n* = 29, *p* = 0.0014] (Figure 2F). Local coherency analysis performed on the SHG signal further confirms the well‐known collagen fiber morphologies found in invasive tumors versus DCIS and healthy tissues (Figure 2G,H). Only a trend can be observed in the SHG^+^ pixel ratio, with a small but not significant increase in invasive tumors compared to healthy and DCIS tissue (Figure S3, Supporting Information).

### 2.3 In the Stroma of Invasive Carcinomas, Tumor Cells Are Predominantly Located in the Vicinity of Untensed Fibronectin Fibers

Since little was known regarding the underpinning physiological processes that lead to the reduction of Fibronectin fiber tension in invasive carcinomas, we next asked where these untensed Fibronectin fibers are located with respect to the tumor cells. As most of our clinical samples came from luminal subtypes carcinomas, we stained for GATA binding protein 3 (GATA3), which is a transcription factor that plays a critical role in the development of epithelial structures in embryonic and adult tissues. In breast carcinomas, this transcription factor is upregulated, with the highest level of expression seen in luminal subtypes.^[^ ^63^ ^]^ As several different cytokeratins, expressed in normal epithelial cells, are also overexpressed in breast carcinoma cells and their detection is prognostic,^[^ ^64^, ^65^ ^]^ an oligoclonal pan‐cytokeratin (panCK) antibody was further used^[^ ^64^ ^]^ in combination with the proliferation marker Ki67 (or MKI67), which varies in abundance across specific breast carcinoma subtypes and thus holds significant prognostic value.^[^ ^66^, ^67^ ^]^ These are typical biomarker combinations used by pathologists as well.

**Figure 3:**
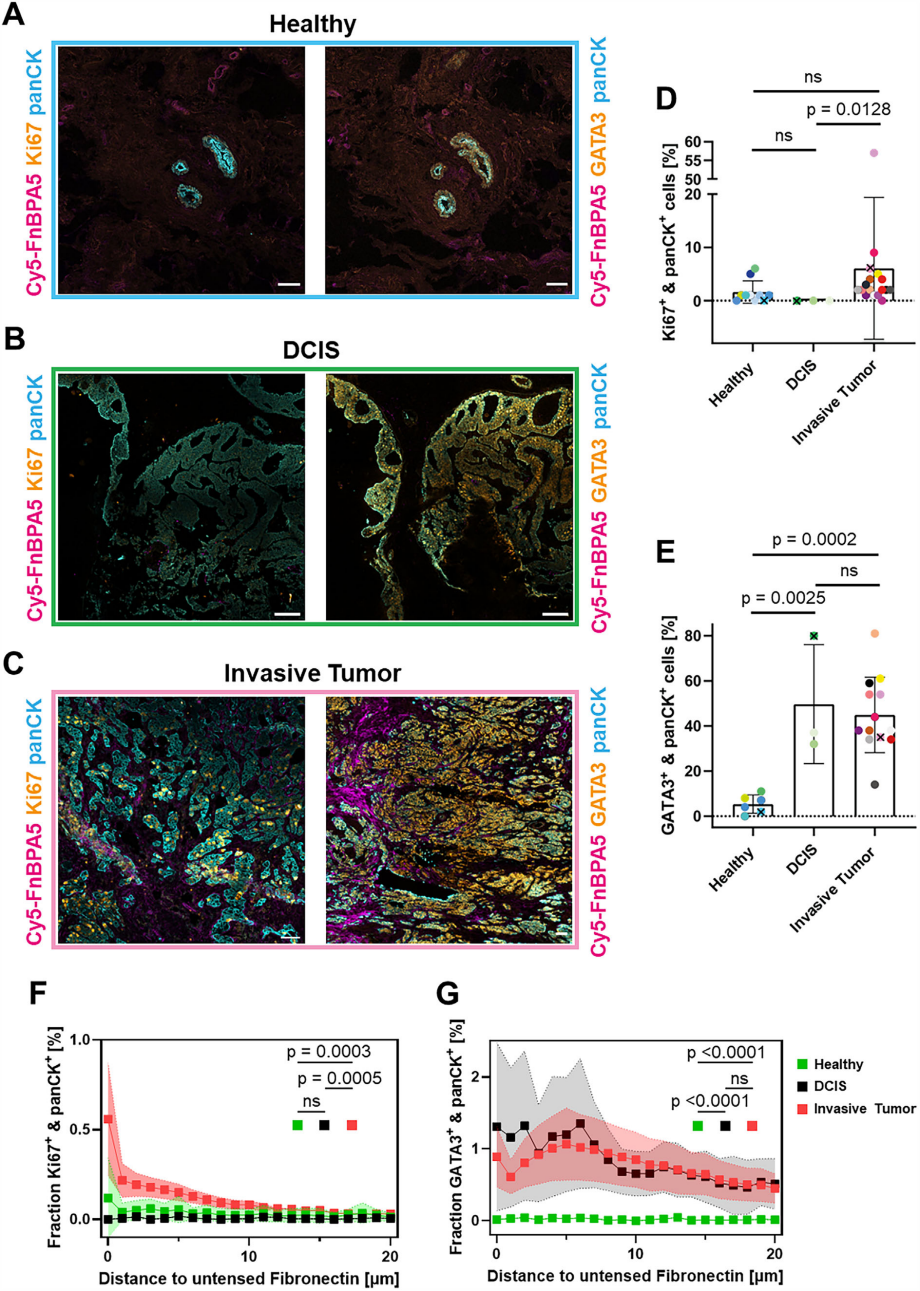
Spatial proximity of cancer cells next to untensed Fibronectin fibers. A–C) Representative confocal images of cryosections stained with pan‐cytokeratin oligoclonal antibody (cyan), a monoclonal antibody against the proliferation marker Ki67 (orange) or a monoclonal antibody against GATA3 (orange), and the Cy5‐FnBPA5 tension probe (magenta), for the following tissues: A) Healthy breast, B) DCIS, and C) Invasive breast carcinoma tissues from patients #6, #11, #28, respectively (Table S1, Supporting Information). D) Percentage of Ki67^+^ & panCK^+^ cells normalized to the total number of cells in each condition as quantified via DAPI staining of cell nuclei. Each dot represents one patient (Healthy: *n* = 10, DCIS: *n* = 4, Invasive Tumor: *n* = 17). Mean ±SD. Kruskal‐Wallis test with Dunn's multiple comparison test. E) Percentage of GATA3^+^ and panCK^+^ cells normalized to the total number of cells in each condition. Each dot represents one patient (Healthy: *n* = 6, DCIS: *n* = 3, Invasive Tumor: *n* = 13). Mean ±SD. One‐way ANOVA with Tukey's multiple comparison test. F,G) For each tissue, the distance between Ki67^+^ & panCK^+^ cells or GATA3^+^ & panCK^+^ cells and Cy5‐FnBPA5^+^ pixels above a threshold is plotted in a distance interval of 1 µm starting from 0 µm, illustrating the significant spatial proximity of tumor cells to untensed Fibronectin fibers. Mean ±SEM. A two‐way ANOVA with Tukey's multiple comparison test was performed to compare the mean cell fractions at all distances (1–20 µm) from untensed Fibronectin fibers across the three groups: healthy, DCIS, and invasive tumors. All images are framed with the patient specific color as defined in Table S1 (Supporting Information). To associate the patient tissues from which the confocal images were taken with the statistical data quantifications, the corresponding colored dots were marked with a cross in each graph. Scale bars: 100 µm. A compilation of images for the different patients is shown in Figure S4 (Supporting Information).

Since the combination of both Ki67 & panCK and GATA3 & panCK markers can also be expressed by healthy cells, a study of tissue morphologies allows us to differentiate between healthy epithelia and cancer‐associated transformed epithelial proliferations (**Figure** **3**A–C). In healthy tissues, GATA3^+^ & panCK^+^ cells are observed, forming circular patterns, characteristic of acini of lobules and ducts.^[^ ^68^ ^]^ Some epithelial cells in the acini are Ki67 positive, indicating proliferative activity in these structures. In DCIS, the panCK signal is sparser than in invasive carcinomas, and the morphology usually resembles that of ducts with a neoplastic expansion of the epithelial compartment.^[^ ^68^ ^]^ In invasive carcinomas, numerous GATA3^+^ & panCK^+^ cells are not organized in circular lobules or ducts, as observed in healthy and DCIS tissues (Figure 3A,B), but are rather organized in streaks with an haphazard and destructive growth (Figure 3C).

As counted by DAPI staining of the nuclei, we quantified the ratio of carcinoma cells to the total cell numbers (Figure 3D,E). In healthy tissues, we find an average of 1.3% of double positive Ki67^+^ & panCK^+^ cells and ≈0% in DCIS. In invasive tumor tissues, which also showed enhanced Cy5‐FnBPA5 binding (Figure 2D), the highest percentages of Ki67^+^ & panCK^+^ cells are found, thus reflecting the higher proliferative capabilities of tumor cells (Figure 3D). Quantifications reveal that healthy samples contain ≈5% of double positive GATA3^+^ & panCK^+^ cells, while DCIS has the highest percentage of GATA3^+^ & panCK^+^ cells at ≈50% compared to 45% for invasive carcinomas (Figure 3E).

To investigate the specific localization of untensed Fibronectin fibers with respect to the tumor cell locations, spatial proximity analyses between Cy5‐FnBPA5^+^ pixels and tumor cells were performed using QuPath.^[^ ^69^ ^]^ This analysis computes the distance between the center of the cell determined with DAPI stains, and the closest Cy5‐FnBPA5^+^ pixel (Figure S3, Supporting Information). In healthy and DCIS tissues, the low proliferative potential is indicated by the close to zero percentage of Ki67^+^ & panCK^+^ cells (Figure 3D), and thus their distance to untensed Fibronectin fibers stays at baseline (Figure 3F). In invasive tumors, Ki67^+^ & panCK^+^ cells are in close contact with untensed Fibronectin fiber pixels, and their presence decreases rapidly with increasing distance from Cy5‐FnBPA5^+^ pixels (Figure 3F). GATA3^+^ & panCK^+^ cells follow a similar correlation with a slower decrease as the distance from untensed Fibronectin fibers increases (Figure 3G), likely due to the higher percentage of GATA3^+^ & panCK^+^ cells when comparing to Ki67^+^ & panCK^+^ cells (Figure 3D,E).

The quantified spatial proximities thus corroborate the observations from the co‐stained images, as nearly no carcinoma cells are found near the sparse untensed Fibronectin fibers in any confocal images of healthy breast tissues (Figure 3A,F,G). In DCIS, the very low Cy5‐FnBPA5 signal is found mostly around the glands and ducts containing proliferative carcinoma cells. In contrast, invasive tumor tissue images display an increased number of carcinoma cells, and proximity analysis revealed that the majority of carcinoma cells are localized within 0–10 µm of Cy5‐FnBPA5^+^ pixels, thus positioning them next to untensed Fibronectin fibers (Figure 3C,F,G).

### 2.4 Significant Presence of α−SMA Expressing CAFs in Regions of Untensed Fibronectin Fibers in Invasive Carcinomas

**Figure 4:**
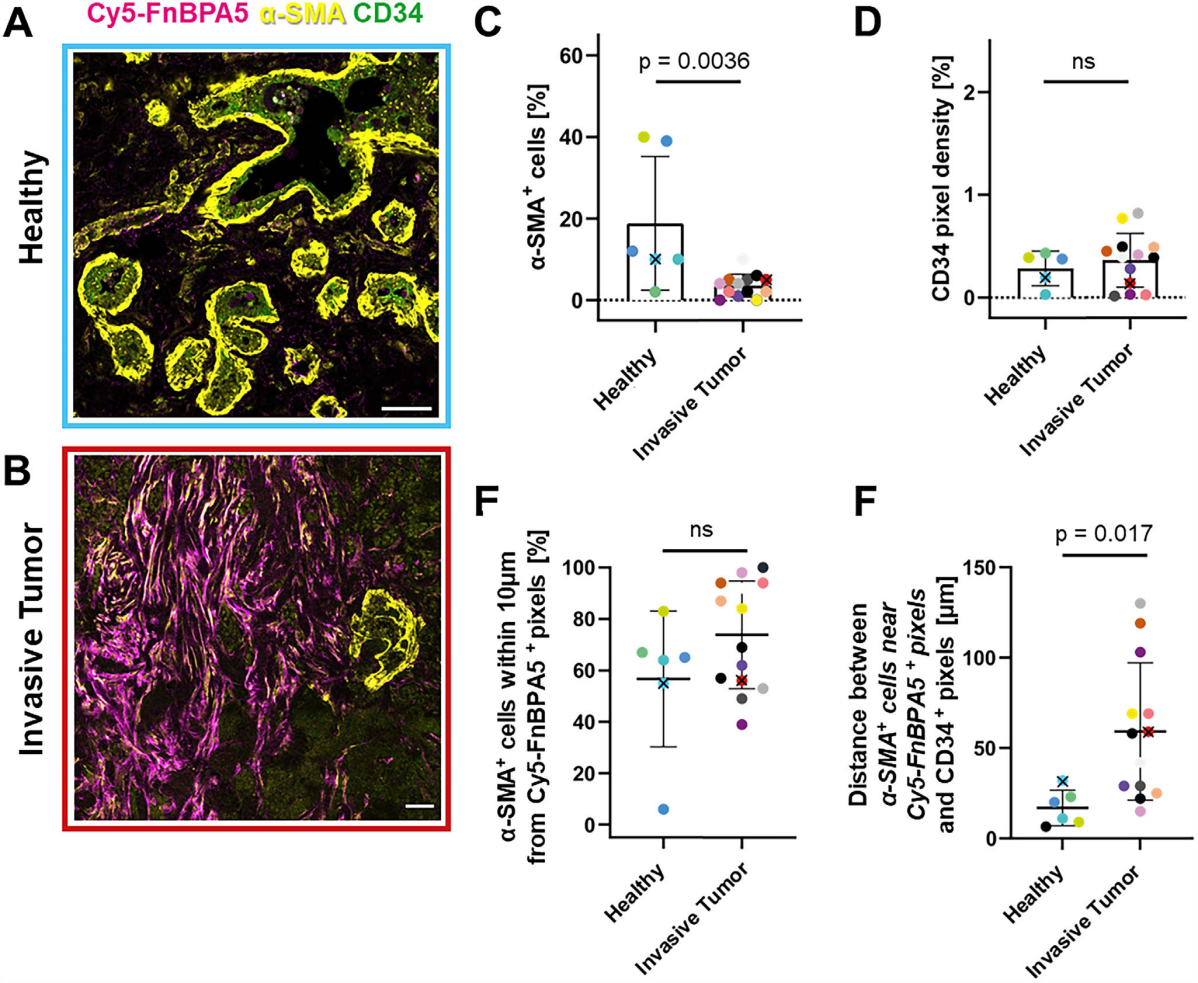
In invasive carcinomas α−SMA positive myofibroblastic CAFs are in close proximity to untensed Fibronectin fibers, away from vessel structures. A) A representative confocal image of healthy breast tissue shows α‐SMA+ pixels (yellow), which can originate from either myoepithelial cells or SMCs, along with the endothelial marker CD34 (green). B) A representative confocal image of a breast carcinoma (pT2) tissue from showing the fiber‐like α‐SMA signal (yellow) in close proximity to Cy5‐FnBPA5 bound Fibronectin fibers (magenta). All images are framed with the patient color, with tissues from patients #6 and #21, respectively (Table S1, Supporting Information). Scale bars: 50 µm. C: Percentage of α‐SMA^+^ cells normalized to the total number of cells in each image as quantified via DAPI stainings of nuclei. D) Density for CD34^+^ pixels above a defined threshold for the CD34 channel normalized to the total pixels of the image. E) Proximity analysis yields the percentage of α‐SMA^+^ cells found within 10 µm of a Cy5‐FnBPA5^+^ pixel. F) Average distance between “α‐SMA^+^ cells that are found within 10 µm of a Cy5‐FnBPA5^+^ pixel” and the nearest CD34^+^ pixel indicative of blood vessels. This shows that α‐SMA^+^ cells found in the vicinity of untensed Fibronectin fibers are on average significantly further away from vessel structures in invasive tumors than in healthy tissues. Each point represents one patient. Colors of data points correlate with patient classification as shown in Table S1 (Supporting Information) (Healthy: *n* = 6, Invasive Tumor: *n* = 14). Mean ±SD. Two‐tailed Student's *t*‐test. To associate the patients from which the confocal images were taken with the statistical data quantifications, the corresponding colored dots were marked with a cross in each graph.

As α‐SMA expressing activated fibroblasts are master regulators of the ECM mechanobiology and central drivers that transform the tumor microenvironment,^[^ ^4^, ^5^, ^6^, ^39^, ^70^ ^]^ we quantified their localization in the breast tissues with respect to untensed Fibronectin fibers, with a focus on invasive carcinomas, excluding DCIS where Fibronectin fiber relaxation was rarely observed. Healthy tissues served as negative controls. Note though that α‐SMA is not only expressed by myofibroblastic CAFs, but also by smooth muscle cells (SMCs) surrounding the blood vessels and myoepithelial cells surrounding the ductal vessels^[^ ^71^ ^]^ and breast lobules.^[^ ^72^ ^]^ Thus, careful phenotyping is necessary to differentiate between α‐SMA produced by CAFs or SMCs. CAFs typically display a fibrous pattern with local alignment, whereas myoepithelial cells and SMC typically line the epithelial cells of the ductal or lobular acini and the endothelial cells of blood vessels, respectively (**Figure** **4**A). In healthy tissues, the α‐SMA signal is found mostly lining the lobular epithelial and endothelial cells, suggesting that it is indeed expressed by contractile myoepithelial cells and SMC^[^ ^72^ ^]^ (Figure 4A). In invasive carcinomas, the majority of the α‐SMA signal is displayed by elongated cell assemblies in proximity to untensed Fibronectin fibers (Figure 4B). In invasive carcinomas, the normalized percentage of α‐SMA^+^ cells is significantly smaller than in healthy breast tissues (Figure 4C).

Performing proximity analysis between α‐SMA^+^ cells and Cy5‐FnBPA5^+^ pixels, no significant differences are observed in the number of α‐SMA^+^ cells located within 10 µm of Cy5‐FnBPA5^+^ pixels when comparing healthy and invasive tumor tissues. This is expected, as α‐SMA is not exclusively expressed by CAFs, as previously mentioned, and untensed Fibronectin fibers are mostly found lining vascular structures in healthy tissues. Therefore, to better distinguish between α‐SMA produced by either SMCs or CAFs, a spatial proximity analysis was performed between “α‐SMA^+^ cells found within 10 µm of Cy5‐FnBPA5^+^ pixels” and vessel structures (CD34^+^ pixels) (Figure 4F). Overall, the density of CD34^+^ pixels remained very similar across all conditions (Figure 4D). In healthy tissues, α‐SMA^+^ cells found in the vicinity of untensed Fibronectin fibers are also in close proximity to CD34^+^ vessels, suggesting that the α‐SMA signal in these tissues most likely originates from SMCs. In contrast, in invasive tumors, the majority of α‐SMA^+^ cells near untensed Fibronectin fibers are located farther from vessel structures (CD34^+^), indicating that α‐SMA is predominantly expressed by CAFs in this context.

The high image‐to‐image heterogeneity in the percentage of α‐SMA^+^ cells reflects the localized presence of lobules and ducts in healthy human breast tissues. In areas predominantly composed of acini, lobules, and ducts, such as the one depicted in Figure 4A, the vast majority of α‐SMA^+^ cells are found lining these vessel structures. Conversely, in regions lacking lobules or ducts, α‐SMA^+^ cells are very sparse.

### 2.5 High‐Grade Invasive Carcinomas Display Enhanced Loss of Fibronectin Fiber Tension, in Contrast to Intermediate‐Grade Invasive Carcinomas

Tumor grading is an important part of tumor diagnosis and treatment, as it serves as a prognostic indicator of tumor aggressiveness and its potential response to adjuvant treatment, as well as a predictive indicator of tumor recurrence.^[^ ^24^ ^]^ Tumor grades range from grade 1 (G1, low‐grade, well‐differentiated, slow‐growing tumors), to grade 3 (G3, high‐grade, poorly differentiated to undifferentiated, and highly proliferative and spreading tumors), where increasing grade reflects increasing tumor aggressiveness and invasiveness. Tumor histologic grading is assessed by characterization and scoring of specific morphologic tumor features such as tubule formation, nuclear pleomorphism and size, and mitotic count.^[^ ^73^, ^74^ ^]^

**Figure 5:**
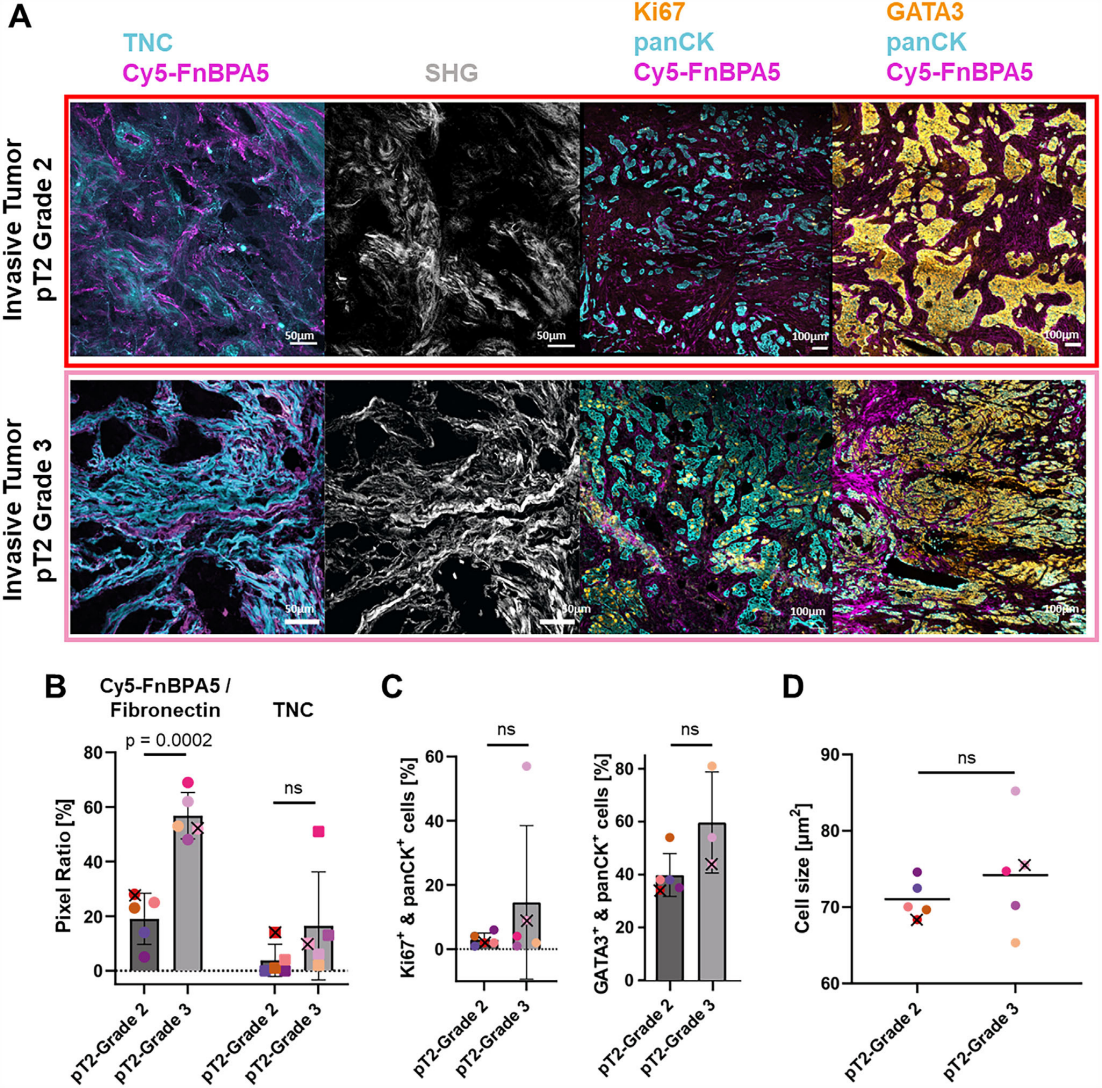
High‐grade pT2 invasive carcinomas show increased Fibronectin fiber relaxation in comparison to intermediate‐grade invasive carcinomas. A) Immunofluorescence of cryosections from grade 2 (*n* = 5) and grade 3 (*n* = 5) pT2 invasive breast carcinomas with antibodies against TNC (cyan), Ki67 (orange) and panCK (cyan), GATA3 (orange) and panCK (cyan). Cryosections were furthermore stained with Cy5‐FnBPA5 (magenta). Dense bundles of collagen fibers detected with SHG (gray) appear aligned in both grade conditions. All images are framed with the patient color from patients #21, #28, respectively (Table S1, Supporting Information). B) Pixel ratio for Cy5‐FnBPA5 gives the percentage of positive pixels above a defined threshold for the Cy5‐FnBPA5 channel (circle), normalized to the total number of positive pixels for the Fibronectin polyclonal antibody. TNC signal was assessed as the percentage of positive pixels above a defined threshold for the TNC channels (square), normalized to the total number of pixels in the studied area. Several cryosections were analyzed for each patient and the average of their respective pixel ratios were plotted. C) Percentage of ki67^+^ & panCK^+^ and GATA3^+^ & panCK^+^ carcinoma cells for each patient (number of patients: *n* = 5, except for pT2‐Grade 3 tumors stained with GATA3‐panCK: *n* = 4). Number of studied areas per patient: *n* = 7. D) Cell size in µm^2^, larger cells are observed in high‐grade tumors (Figure S6, Supporting Information). Each color represents one patient (number of studied areas per patient: *n* = 3). Mean ±SD. Two‐tailed Student's *t*‐test. To associate the patients from which the confocal images were taken with the statistical data quantifications, the corresponding colored dots were marked with a cross in each graph.

For invasive tumors of both grades (G2 and G3), representative confocal images of TNC and Cy5‐FnBPA5 staining show an enhanced fraction of TNC^+^ and Cy5‐FnBPA5^+^ pixels (**Figure** **5**A). Cy5‐FnBPA5 signals are significantly increased by ≈200% in high‐grade tumors compared to intermediate grades (Figure 5B). A strong SHG signal is detected in both grades, revealing numerous collagen fiber bundles in proximity to Cy5‐FnBPA5^+^ pixels (Figure 5A). High‐grade carcinomas tend to have an increased tumor cell density and cell size (Figure 5C,D; Figure S6, Supporting Information), in agreement with the literature, where tumor cells are expected to be more proliferative, compared to intermediate‐grade tumors.^[^ ^74^ ^]^ These data show for the first time that tumor grade in invasive human breast cancers correlates with an enhanced presence of untensed Fibronectin fibers, with Fibronectin fibers undergoing progressive relaxation as tumor grade increases.

### 2.6 In the Stroma of Invasive Carcinomas, More than 60% of Cytotoxic T Cells and Regulatory T Cells (Tregs) Are Found in Proximity to Untensed Fibronectin Fibers

**Figure 6:**
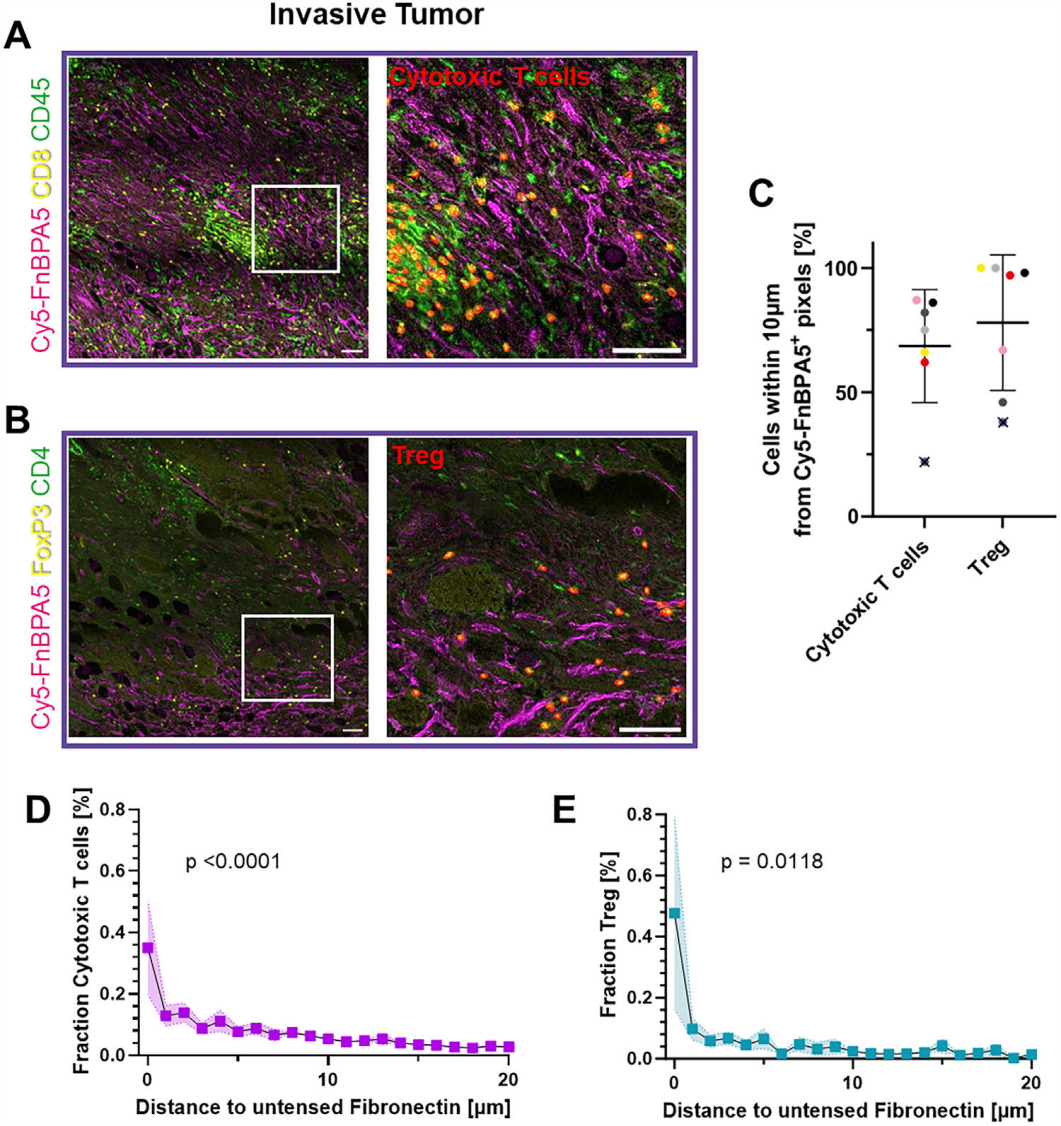
In invasive tumors, T cells are preferentially found in proximity to untensed Fibronectinfibers. A) Immunofluorescence images of cryosections of invasive breast carcinoma stained with antibodies against CD8 (yellow), CD45 (green), and FnBPA5 (magenta). B) Immunofluorescence images of cryosections of invasive breast carcinoma stained with antibodies against FoxP3 (yellow), CD4 (green), and FnBPA5 (magenta). Cytotoxic T cells (CD45^+^ & CD8^+^) or Treg (Foxp3^+^ & CD4^+^) are highlighted in red in the zoom‐in images, showing the proximity between the respective immune cells and untensed Fibronectin fibers (magenta). All images are framed with the patient color, patient #23 (Table S1, Supporting Information). Scale bar: 100 µm. C: Spatial proximity analysis of T cells with respect to the Cy5‐FnBPA5^+^ pixels displaying the percentage of Cytotoxic T cells (CD45^+^ & CD8^+^) and regulatory T cells (CD4^+^ & FoxP3^+^) that are found within 10 µm of Cy5‐FnBPA5^+^ pixels. Each dot represents one patient (*n* = 7). Mean ±SD. D–E: The distance between Cytotoxic T cells (E) or Treg (D) and Cy5‐FnBPA5^+^ pixels above a threshold is plotted in a distance interval of 1 µm starting from 0 µm, illustrating the spatial proximity of tumor cells to untensed Fibronectin fibers. Mean ±SEM. One‐way ANOVA was performed to show that the total cell fractions varied significantly with increasing distance from untensed Fibronectin fibers. To associate the patients from which the confocal images were taken with the statistical data quantifications, the corresponding colored dots were marked with a cross in each graph.

In murine models, it was previously found that tumor matrix tracks, rich in TNC and untensed Fibronectin fibers, play a role in the immunomodulation of the tumor microenvironment by entrapping immune cells.^[^ ^57^, ^75^ ^]^ Representative confocal images stained for cytotoxic T cells (CD45^+^ & CD8^+^) and regulatory T cells (Tregs) (CD4^+^ & FoxP3^+^) in invasive human carcinomas now show close proximity between both immune cell types and FnBPA5‐positive Fibronectin fibers (**Figure** **6**A,B). Proximity analysis reveals that the majority of Cytotoxic T cells and Treg cells are found in the vicinity of untensed Fibronectin fibers, with on average 68% of cytotoxic T cells (CD45^+^ & CD8^+^) and 77% of Treg (CD4^+^ & FoxP3^+^) being within 10 µm of untensed Fibronectin fiber as depicted in Figure 6C. The fraction of cytotoxic T cells and Tregs significantly varied with increasing distance from untensed Fibronectin fibers, as indicated by one‐way ANOVA (*p *< 0.0001, *p* = 0.0118, respectively) (Figure 6D,E). These close proximities tend to decrease rapidly (Figure 6D,E). A trend analysis (one‐way ANOVA, test for trend) revealed a significant pattern across distances (*p* < 0.0001 and *p* = 0.0012 for cytotoxic T cells and Tregs, respectively), confirming a systematic decrease in immune cell presence with increasing distance from the Cy5‐FnBPA5 signal.

## 3 Discussion

The tumor microenvironment is increasingly targeted by drug and immune therapies,^[^ ^18^, ^76^ ^]^ as it undergoes major compositional, structural, and mechanical changes during tumor growth and progression.^[^ ^3^, ^6^, ^12^, ^18^ ^]^ The composition and organization of ECM fibers, including various collagens, TNC and Fibronectin among others, can contribute to immunosuppressive tumor microenvironments and reduce the cancer cell responsiveness to therapy, partly by acting as a barrier to therapeutic agents.^[^ ^6^, ^7^, ^49^, ^57^, ^77^ ^]^ This pathological ECM remodeling is orchestrated by the reciprocal crosstalk between stroma, cancer and immune cells, and the progressively transformed ECM that surrounds them.^[^ ^13^, ^78^, ^79^, ^80^ ^]^ Most previous studies explored the physical traits of cancer by focusing on tissue stiffness, predominantly mapped by indentation.^[^ ^20^, ^24^, ^81^, ^82^, ^83^ ^]^ Breast tissue stiffness gradually increases with tumor malignancy and promotes tumor cell migration, which may be critical for tumor development toward an invasive phenotype.^[^ ^15^, ^17^, ^45^, ^48^, ^84^ ^]^ Many factors contribute to increased tumor stiffness, including the upregulated expression of ECM proteins, collagen fiber alignment and crosslinking, such as by lysyl oxidase (LOX),^[^ ^15^, ^47^ ^]^ and the enhanced contractility of myofibroblastic CAFs^81^. Conversely, interfering with LOX‐induced collagen fiber crosslinking reduced the ECM content and tumor stiffness, leads to improved T‐cell migration and increased efficacy of anti‐PD‐1 blockade.^[^ ^85^ ^]^ While measurements of tissue stiffness have been introduced as clinical indicators to detect tumors, nothing was previously known about the tensional state of individual ECM fibers in human tissues. Using our peptide tension probe FnBPA5, whose nm affinity to Fibronectin fibers is destroyed by fiber stretching,^[^ ^51^, ^56^, ^57^ ^]^ we discovered here that Fibronectin fibers have partially lost their tension in invasive human tumor stroma in locations that are in close proximity to α−SMA positive myofibroblastic CAFs, as well as T‐cells and cancer cells, whereas they are still mostly tensed in DCIS and in healthy breast tissues (Figures 1, 2). The tumor stroma, enriched in untensed Fibronectin fibers, straightened collagen fiber bundles, and TNC, surrounds tumor nests that containing the majority of tumor cells (Figures 1, 2).

The main distinction between the precursor DCIS and invasive carcinomas is the loss of the myoepithelial layer and basement membrane, which then allows tumor cells to escape the ductal areas and migrate into adjacent tissues.^[^ ^28^ ^]^ In invasive tumors, the invading CAFs progressively pull on and align nearby ECM fibers, resulting in the well‐characterized collagen fiber signature of invasive tumors.^[^ ^27^, ^28^, ^49^, ^81^ ^]^ In contrast, in DCIS, the tumor still grows by compressing the surrounding tissue as cancer cells cannot escape the tumor nest, keeping the same tensional signature as healthy tissues, with mostly wavy collagen fibers and stretched Fibronectin fibers (Figure 2). While some straightening of collagen fibers perpendicular to the tumor boundary, as described in the literature as tumor‐associated collagen signature 3 (TACS‐3), can be observed in some DCIS cases, in most patients, these collagen fibers maintain a wavy appearance with minimal linearization.^[^ ^24^, ^28^, ^29^ ^]^ These findings are also corroborated by our data, as in our four DCIS patients (numbers 11–14), the collagen fibers retain a wavy appearance with limited straightening (Figure S1, Supporting Information). The differences in Cy5‐FnBPA5 binding compared to healthy breast tissue are within statistical error as confirmed by ratiometric analysis (Figure 2). The finding that DCIS tissues exhibit a Fibronectin fiber tensional signature similar to that of healthy breast tissues may be due to the fact that DCIS grows within defined borders. Therefore, their surrounding ECM carries little fibrotic signatures, which is consistent with the low presence of TNC (Figure 2). Upon the onset of cancer cell invasion (Figure 2), collagen fibers get progressively aligned perpendicular to the duct borders as migrating cells pull on them.^[^ ^24^, ^27^, ^28^, ^49^ ^]^ Our data now suggest that upregulated collagen fiber assembly, straightening, and alignment correlate with major local reductions of Fibronectin fiber tension (Figure 2). Although our observations suggest that Fibronectin fiber relaxation is largely absent in DCIS, we cannot yet say whether Fibronectin fiber relaxation precedes tumor invasion into the surrounding matrix, or is a consequence of invasion. Nevertheless, the relaxation of Fibronectin fibers correlates, and might even be a characteristic mechano‐chemical signature for cancer progression, as high‐grade invasive carcinomas, which are associated with increased tumor aggressiveness and invasiveness, are richer in untensed Fibronectin fibers than intermediate‐grade invasive carcinomas (Figure 5).

Untensed Fibronectin fibers are in spatial proximity to well‐recognized hallmarks of cancer, including myofibroblastic CAFs, TNC, and thick bundles of aligned collagen fibers^[^ ^48^ ^]^ (Figures 2, 4). The observed proximity of untensed Fibronectin fibers to α−SMA positive CAFs might be counter‐intuitive at first glance, as α‐SMA expressing activated fibroblasts are more contractile than their nonactivated counterparts. One hypothesis could be that TNC plays a crucial role in promoting the relaxation of Fibronectin. TNC, which has been associated with the recruitment and maturation of α−SMA positive CAFs,^[^ ^86^ ^]^ is also known for its anti‐adhesive properties. Yet, Fibronectin fiber relaxation is still seen in tumors of TNC‐KO mice, suggesting that TNC expression is not required for Fibronectin fiber relaxation to occur.^[^ ^57^ ^]^ Several other mechanisms may contribute to the loss of fibronectin tension. First, as single Fibronectin fibers are far more extensible than collagen fibers,^[^ ^31^ ^]^ the contractile α−SMA positive CAFs might switch to pulling preferentially on the sturdier collagen fiber bundles than on Fibronectin fibers, as shown already in vitro,^[^ ^36^ ^]^ thereby reducing Fibronectin's fiber tension. Second, activated stroma contains elevated levels of proteases, including MMPs^3^. These proteases orchestrate ECM remodeling and, when imbalanced, promote tumor growth and progression.^[^ ^45^, ^78^, ^84^ ^]^ The increased proteolytic remodeling activity of the activated stroma could thus also contribute to enhanced Fibronectin fiber cleavage. This could not only locally cleave Fibronectin fibers, but also release Fibronectin fragments, called matrikines, some of which have physiological roles that differ from the full‐length molecule.^[^ ^87^ ^]^ Regardless of whether Fibronectin fibers are cleaved or kept under low cellular tension by losing their load‐bearing function, untensed Fibronectin fibers display different molecular binding sites than highly stretched fibers.^[^ ^32^ ^]^ Importantly, this switch in Fibronectin's displayed biochemistry^[^ ^32^ ^]^ is a direct consequence of fibroblast activation toward a myofibroblastic phenotype^[^ ^27^, ^47^ ^]^ and occurs concomitant with active stroma remodeling in humans and various mouse tumor models.^[^ ^56^, ^57^ ^]^ The appearance of Fibronectin fibers that progressively lose tension scales with increasing grade of invasive carcinoma.

The functional significance of the progressive loss of Fibronectin fiber tension is multifaceted. Stretching or relaxing ECM fibers can tune Fibronectin's interactions with molecular binding partners, such as integrins, enzymes, growth factors and cytokines, by either opening otherwise cryptic binding sites, or by destroying binding motifs.^[^ ^32^ ^]^ In addition, we found an increased ratio of α_5_β_1_ versus α_v_β_3_ integrins in our invasive tumors compared to the healthy controls (Figure S7, Supporting Information), which is important since integrin α_5_β_1_ recognizes both Fibronectin's RGD loop on FnIII_10_ and its synergy site on FnIII_9_. Importantly, integrin α_5_β_1_ affinity to Fibronectin is regulated by fiber stretching, where untensed Fibronectin fibers exhibit a higher affinity for α_5_β_1_ versus α_v_β_3_ integrins than stretched fibers.^[^ ^33^ ^]^ This aligns with previous SMD predictions illustrating that Fibronectin stretching increases the distance between the synergy site and the RGD loop.^[^ ^88^ ^]^ Taken together, we would like to propose that untensed Fibronectin fibers might thus be more potent to activate integrin α_5_β_1_, than stretched fibers. As integrin engagement with the ECM can potentially also tune integrin trafficking with concomitant changes in cell functions,^[^ ^89^, ^90^ ^]^ the enhanced presence of untensed Fibronectin fibers could tune integrin α_5_β_1_ internalization and thereby the local cell behavior. Enhanced α_5_β_1_ integrin recycling correlates with pseudopod extension and cell invasion, at least for human ovarian cancer cell lines in Fibronectin‐enriched 3D collagen matrices.^[^ ^90^, ^91^ ^]^

Previous data further suggested that mechanical forces can self‐tune the composition of ECM‐binding partners that are harbored or even stored inside the stroma,^[^ ^32^, ^92^ ^]^ or impact the accessibility of proteolytic cleavage sites as previously shown for collagen fibers.^[^ ^93^ ^]^ TG2 and IL‐7 are two of Fibronectin's binding partners, whose binding affinity is tuned by Fibronectin fiber stretching.^[^ ^35^, ^40^ ^]^ Both are known to play particularly important roles in the pathology of invasive tumors and are serving as drug targets.^[^ ^42^, ^43^, ^94^ ^]^ We recently found that the binding of TG2 in its closed conformation is upregulated when Fibronectin fibers lose their tension.^[^ ^35^ ^]^ This is significant in the context of cancer, as the crosslinking enzyme TG2 plays significant roles in ECM remodeling. At least in vitro, poorly migratory breast cancer cells can be turned metastatic by activating fibroblasts via TG2‐containing microvesicles.^[^ ^95^ ^]^ We would like to propose that the relaxation of Fibronectin fibers as described here might thus contribute to the accumulation of TG2 in the stroma of invasive cancers, thereby influencing tumor initiation, growth, and metastasis.^[^ ^43^ ^]^ The immune‐boosting properties of IL‐7 are also well recognized, yet IL‐7 binds less to untensed Fibronectin fibers.^[^ ^40^ ^]^ Our finding that invasive tumors show a significantly enhanced amount of untensed, rather than stretched Fibronectin fibers is significant, as IL‐7 is far more potent to promote T‐, B‐ and dendritic cell maturation and function in a Fibronectin‐bound state.^[^ ^42^ ^]^ Taken together, this suggests that the stroma of invasive tumors can retain less Fibronectin‐bound IL‐7, and through this mechano‐regulated mechanism could contribute to rendering the cancer stroma more immunosuppressive. Knowledge of such mechano‐regulated binding mechanisms is crucial, as the community explores currently whether IL‐7 can serve as a promising adjuvant ensuring effective T cell responses and memory in combination with cancer vaccines,^[^ ^94^ ^]^ and whether immunocytokines can strengthen anti‐PD‐(L)1 therapy by promoting T‐cell survival.^[^ ^96^ ^]^ The possibility that untensed Fibronectin fibers are immunosuppressive is further supported by their high proximity to leukocytes, as previously also shown in a mouse model.^[^ ^57^ ^]^ Our data indicated that untensed Fibronectin fibers may sequester the majority of cytotoxic T cells and Treg cells (Figure 6), potentially hindering their infiltration into tumor nests. This may also reduce the efficiency of contact‐dependent lysis of tumor cells and facilitate tumor cell escape from immune surveillance. Taken together, the finding of a progressive loss of Fibronectin fiber tension in invasive tumors suggests that alterations of their tensional state might play an unrecognized role in steering tumor progression, cell migration, and T cell activation.^[^ ^82^, ^97^ ^]^ Upstream of the well‐recognized players orchestrating mechanosensing and mechanotransduction, such as integrins, cadherins, mechano‐gated ion channels, G protein‐coupled receptors and various transcription factors, the mechanical strain of ECM proteins may play a significant role in the mechano‐chemical shift of the cancer stroma and the signaling molecules harbored within. These phenotypic adaptations to altered mechanical ECM properties might get imprinted in the resulting shift of cell phenotypes through persistent epigenetic changes.^[^ ^9^ ^]^

Even though cancer cell‐centric approaches had traditionally been taken in oncology, a major paradigm shift is underway with the development of tumor stroma targeting therapies, such as immunotherapies.^[^ ^76^ ^]^ The intimate reciprocal crosstalk between CAFs and the ECM they deposit leads to significant remodeling in the tumor stroma that shapes nearly all stages of tumorigenesis and progression. A thorough understanding of how cell‐generated mechanical forces can switch the biochemical display of ECM fibers is therefore critical for the development of novel strategies to target the stroma. The discovery of a prominent presence of untensed Fibronectin fibers in invasive tumors, which increases with tumor progression, and their proximity to cancer cell nests, α−SMA positive CAFs, and particularly immune cells is of paramount importance, as the physical signature of individual ECM fibers might play so far unrecognized roles in tumor progression, as well as immune cell silencing or sequestration. Fibronectin fibers, which harbor many binding sites for growth factors, cytokines, crosslinking and proteolytic enzymes, and other matrix‐bound molecules, are crucial components of the remodeled tumor microenvironment. Alterations in the tensional state of Fibronectin fibers, and thus in their mechano‐chemistry, can switch the structure‐function relationship of many of its domains.^[^ ^32^ ^]^ We thus propose that tuning Fibronectin fiber tension allows cells to modulate the bioavailability of Fibronectin's binding partners in the stroma. In a mechanically driven reciprocal feedback loop, this will alter outside‐in cell signaling by tuning various mechanotransduction pathways, mechanically and biochemically. This process may ultimately contribute to tumor progression, further highlighting the role of ECM proteins as mechano‐chemical signal converters beyond their structural functions.

Our discovery is prone to stimulate the search for mechanical biomarkers to target cancer stroma, as well as for developing novel stroma‐targeting therapies that target not only a specific molecule of interest, but also its physical state. Anti‐cancer treatments or immunotherapies could be delivered using peptides with multivalent binding motifs, such as FnBPA5, to specifically target the mechanically transformed stroma, as previously shown already in a mouse model,^[^ ^51^ ^]^ as this stroma surrounds cancer cell nests and harbors the often immunosuppressed immune cells. Physical ECM fiber signatures may thus play so far unrecognized roles in promoting invasiveness, as well as in immune cell silencing or sequestration, and could be targeted for drug delivery and immunotherapy.

## 4 Experimental Section

### Human Breast Biopsy Sampling and Characterization

Healthy and tumor tissues from human breasts were obtained from patients by the Pathology Department of Kantonspital Baden (KSB) and from the Tissue Bank Bern (TBB) in accordance with our Ethical approval (BASEC 2017‐01828). Informed written consent was obtained from each patient prior to surgical resection of breast tissue. Cryosections were realized by technicians and pathologists of the Pathology Department of Kantonspital Baden after macroscopic and microscopic diagnosis and by pathologists of TBB, and stored at − 80 °C until further use. Further characterization of the resected tissues as shown in Table S1 (Supporting Information) was performed by pathologists from KSB and TBB. Healthy tissue was either from a breast reduction surgery or from tissue adjacent to the tumor and classified as healthy by pathologists (Table S1, Supporting Information).

### Immunofluorescence (IF) of Human Patients Tissue Sections

Cryopreserved tissue sections from both healthy and tumor tissues were stained for specific ECM markers and for the Cy5‐FnBPA5 tension probe following the previously developed protocol.^[^ ^51^, ^56^, ^57^ ^]^ Briefly, cryosections were first blocked 30 min with 4% bovine serum albumin (BSA) before being incubated for 1 h at room temperature with 5 µg mL⁻^1^ of Cy5‐FnBPA5 or Cy5‐labeled scrambled‐FnBPA5 as control. Sections were further washed and fixed with 4% paraformaldehyde (PFA) in 1xPBS for 10 min. For intracellular markers’ detection, the sections were permeabilized with 0.1% Triton X‐100 in 1xPBS for 30 min. Tissue sections were further blocked with 5% goat serum with 0.3 m glycine for 1 h and later incubated with primary antibodies listed in **Table** **2** overnight at 4 °C. Secondary antibodies listed in Table 2 were then applied for 1 h at room temperature, and some sections were further stained with DAPI 2 µg mL⁻^1^ for 10 min before being mounted with a hardening DAKO Fluorescence mounting medium (DAKO, Denmark). The stained and mounted slides were imaged after 24 h using a confocal microscope (Leica SP8).

**Table 2:** List of antibodies used.

Antibody	Clone	Reference, Company
**Ki67**	Polyclonal	ab15580, Abcam
**pan cytokeratin (panCK)**	AE1/AE3 + 5D3	ab86734, Abcam
**GATA3**	GATA3‐ChIP Grade	ab199428, Abcam
**α‐SMA**	1A4	ab7817, Abcam
**Fibronectin**	Polyclonal	ab23750, Abcam
**TNC**	B28.13	Provided by G.Orend lab
**CD34**	EP373Y	ab81289, Abcam
**CD8**	C8/144B	ab17147, Abcam
**CD45**	EP322Y	ab40763, Abcam
**CD4**	EPR6855	ab133616, Abcam
**FoxP3**	236A/E7	ab20034, Abcam
**α_v_**	P2W7	ab11470
**α_5_**	EPR7854	ab150361
**Goat anti‐Rabbit alexa fluor 488**		A‐11034, Invitrogen, ThermoFisher Scientific
**Goat anti‐Mouse alexa fluor 555**		A‐21424, Invitrogen, ThermoFisher Scientific

### Confocal and Multi‐Photon Imaging of IF‐Stained Tissue Sections

Stained cryosections were imaged with a Leica SP8 confocal microscope, equipped with a multiphoton laser for SHG imaging. Full tissue cryosections overviews were acquired with a 10x objective, and further zoom‐in of specific areas was done with higher resolution, using a 20x objective. Multiple high‐resolution images were acquired from each sample. SHG images were acquired with a 25x water objective.

### Image Analysis: Quantification of Pixel Densities

Confocal images of full tissue sections were analyzed with Qupath (version 0.4.2).^[^ ^69^ ^]^ To exclude noise and artifacts caused by tissue detachment during IF, the full tissue sections were carefully manually annotated. For all channels, training images were generated and pixel classification was performed based on the manual fiber/signal annotations. After successful training, the classifier was then applied to the tissue sections and positive pixel masks were created for both channels. The area covered by the masks was then counted and expressed as a percentage of the total tissue section area.

For Cy5‐FnBPA5, a ratiometric analysis was performed to normalize Cy5‐FnBPA5 positive pixels with respect to polyclonal Fibronectin antibody pixels which stained all Fibronectin irrespective of its tensional state.

### Ratiometric Image Analysis to Visualize Untensed versus Tensed Fibronectin Fibers

Ratiometric image analysis of Cy5‐FnBPA5 staining relative to total Fibronectin was performed using a custom program written in MATLAB (Natick, MA, USA). Briefly, images were thresholded using Otsu's method, and masked based on where both Fibronectin and Cy5‐FnBPA5 signals passed the threshold. The fluorescence intensities of Cy5‐FnBPA5 versus total Fibronectin were calculated for each pixel and mapped using a color scale based on the ratios.

### Coherency Analysis of Collagen Fibers (SHG Positive)

From the SHG images, collagen fibers were identified using Frangi vesselness^[^ ^98^ ^]^ followed by fiber orientation analysis using OrientationJ in ImageJ.^[^ ^99^ ^]^

### Quantifications of Cancer Cell Density and Size

Cancer cell detection was performed using Qupath (version 0.4.2).^[^ ^69^ ^]^ To eliminate noise and artifacts caused by tissue detachment during IF, the entire tissue sections were carefully manually annotated. Cancer cells were labeled using two sets of antibodies, either Ki67 with panCK or GATA3 with panCK. Using the built‐in function “cell detection”, cells were identified based on their DAPI signal. Then, to classify each cell, a threshold was created based on the cell mean intensity value for each marker using a single measurement classifier. Finally, marker colocalization was performed by creating composite classifiers based on either the combination of Ki67 and panCK or GATA3 and panCK single measurement classifiers. The results were plotted using GraphPad Prism (version 10.1.2) as the percentage of Ki67^+^ & panCK^+^ or GATA3^+^ & panCK^+^ cells. The built‐in function “cell detection” of Qupath allows us to calculate the size of each cell based on the size of the nucleus.

### Proximity Analyses between Either Cancer or T Cells and Untensed Fibronectin Fibers (Cy5‐FnBPA5^+^)

Confocal images of zoom‐in areas of cryosections stained with the Cy5‐FnBPA5, DAPI, and with antibodies against panCK, Ki67, and GATA3 were processed in Qupath.^[^ ^69^ ^]^ To eliminate noise and artifacts caused by tissue detachment during IF, the entire tissue sections were carefully manually annotated. The detection of untensed/relaxed Fibronectin fibers was based on a machine learning approach. To train the model, several training images were randomly selected on zoom‐in areas of cryosections stained with Cy5‐FnBPA5. For each class, negative (non‐relaxed Fibronectin fibers) and positive (relaxed Fibronectin fibers), manual annotations were performed until the model was successfully able to recognize relaxed Fibronectin fibers. After successful training of the model, the classifier was then applied to tissue sections, and masks with positive pixels for relaxed Fibronectin fibers were created. Cancer cells were classified as described above. Finally, the proximity analysis between cancer cells and relaxed Fibronectin fibers was performed using Qupath's built‐in function “spatial analysis‐ distance to annotations 2D”, which calculates the smaller distance between cells and Cy5‐FnBPA5 positive pixels. This analysis computes the distance between the center of the cell nucleus as determined with DAPI stain, and the closest positive pixel of Cy5‐FnBPA5 (Figure S3, Supporting Information). A similar approach was used to calculate the proximity analysis between α‐SMA^+^ cells to CD34 signal and relaxed Fibronectin, as well as the proximity analysis between immune cells (CD8^+^ T and Regulatory T cells) and relaxed Fibronectin fibers.

### Statistical Analysis

Statistical analyses were performed using GraphPad Prism 10.1.2. Parametric or non‐parametric distribution was assessed based on four normality tests, D'Agostino‐Pearson, Anderson‐Darling, Shapiro‐Wilk, and Kolmogorov‐Smirnov, and based on QQplot graphical assessment as provided by GraphPad Prism. Statistical significance of two parametric groups was performed using the unpaired Student's *t* test, while two non‐parametric groups were analyzed using the Mann–Whitney test. Comparisons of three or more equally distributed groups were compared using one‐way ANOVA with Turkey's multiple comparison test and non‐parametric equivalents were subjected to Kruskal‐Wallis test with Dunn's multiple comparison test.

For the spatial proximity analysis between cancer cells and untensed Fibronectin fibers, a two‐way ANOVA with Tukey's multiple comparison test was performed to compare the mean cell fractions at all distances (1–20 µm) from untensed Fibronectin across the three groups: healthy, DCIS, and invasive tumor. For the spatial proximity analysis between immune cells and untensed Fibronectin fibers, a one‐way ANOVA was performed to show that total cell fractions varied significantly with increasing distance from untensed Fibronectin fibers, followed by a test for trend (one‐way ANOVA, test for trend) to test for the presence of a significant pattern between immune cells presence and distance from untensed Fibronectin fibers.

## Conflict of Interest

Viola Vogel is a co‐founder of Tandem Therapeutics AG (CHE‐192.070.977), an early‐state start‐up aiming to develop extracellular matrix targeting drugs. The other authors declare no competing interests.

## Author Contributions

A.M. and C.M.F. contributed equally to this work. G.S. and V.V. share last authorship. A.M. initiated the collaboration with TBB, data curation, formal analysis, investigation, visualization, and writing and editing of the manuscript. C.M.F. initiated the collaboration with KSB, data curation, conceptualization, formal analysis, investigation, visualization, and writing – original draft. C.L. carried out patient recruitment, breast tissue sample resections, and writing study protocol and helping with ethical approval. L.C. carried out tissue diagnostics. J.G. initiated the collaboration with KSB and helping with ethical approval. G.S. carried out tissue diagnostics, writing study protocol and ethical approval, and providing insights and inputs into the pathological aspect and correlation to histopathological findings. V.V. initiated the collaboration with KSB and TBB, helping to write the study protocol and acquiring ethical approval, conceptualization, funding acquisition, formal analysis, and writing and editing of the manuscript. All authors have seen and approved the manuscript.

## Supporting information



Supporting Information

## Data Availability

The data that support the findings of this study are available from the corresponding author upon reasonable request.

## References

[1] H. Sung , J. Ferlay , R. L. Siegel , M. Laversanne , I. Soerjomataram , A. Jemal , F. Bray , CA Cancer J. Clin. 2021, 71, 209. 33538338 10.3322/caac.21660

[2] R. Malik , P. I. Lelkes , E. Cukierman , Trends Biotechnol. 2015, 33, 230. 25708906 10.1016/j.tibtech.2015.01.004 PMC4380578

[3] J. Winkler , A. Abisoye‐Ogunniyan , K. J. Metcalf , Z. Werb , Nat. Commun. 2020, 11, 5120. 33037194 10.1038/s41467-020-18794-x PMC7547708

[4] G. Biffi , D. A. Tuveson , Physiol. Rev. 2021, 101, 147. 32466724 10.1152/physrev.00048.2019 PMC7864232

[5] M. H. Sherman , M. P. di Magliano , Annu. Rev. Cancer Biol. 2023, 7, 43.

[6] E. Henke , R. Nandigama , S. Ergün , Front. Mol. Biosci. 2020, 6, 160. 32118030 10.3389/fmolb.2019.00160 PMC7025524

[7] J. P. Wang , A. Hielscher , J. Cancer 2017, 8, 674. 28367247 10.7150/jca.16901 PMC5370511

[8] S. Wang , J. Li , Y. Zhao , Cancer Pathogenesis and Therapy 2024. 10.1016/j.cpt.2023.11.005 PMC11252504 39027145

[9] E. Cambria , M. F. Coughlin , M. A. Floryan , G. S. Offeddu , S. E. Shelton , R. D. Kamm , Nat. Rev. Cancer 2024, 24, 216. 38238471 10.1038/s41568-023-00656-5 PMC11146605

[10] H. T. Nia , L. L. Munn , R. K. Jain , Science 2020, 370, aaz0868. 10.1126/science.aaz0868 PMC8274378 33122355

[11] S. Safaei , R. Sajed , A. Shariftabrizi , S. Dorafshan , L. Saeednejad Zanjani , M. Dehghan Manshadi , Z. Madjd , R. Ghods , Cancer Cell Int. 2023, 23, 143. 37468874 10.1186/s12935-023-02992-w PMC10357884

[12] B. Piersma , M.‐K. Hayward , V. M. Weaver , Biochim. Biophys. Acta (BBA) – Rev. Cancer 2020, 1873, 188356. 10.1016/j.bbcan.2020.188356 PMC7733542 32147542

[13] F. L. Miles , R. A. Sikes , Mol. Cancer Res. 2014, 12, 297. 24452359 10.1158/1541-7786.MCR-13-0535 PMC4066664

[14] D. T. Butcher , T. Alliston , V. M. Weaver , Nat. Rev. Cancer 2009, 9, 108. 19165226 10.1038/nrc2544 PMC2649117

[15] K. R. Levental , H. Yu , L. Kass , J. N. Lakins , M. Egeblad , J. T. Erler , S. F. T. Fong , K. Csiszar , A. Giaccia , W. Weninger , M. Yamauchi , D. L. Gasser , V. M. Weaver , Cell 2009, 139, 891. 19931152 10.1016/j.cell.2009.10.027 PMC2788004

[16] O. Chaudhuri , J. Cooper‐White , P. A. Janmey , D. J. Mooney , V. B. Shenoy , Nature 2020, 584, 535. 32848221 10.1038/s41586-020-2612-2 PMC7676152

[17] M. W. Conklin , J. C. Eickhoff , K. M. Riching , C. A. Pehlke , K. W. Eliceiri , P. P. Provenzano , A. Friedl , P. J. Keely , Am. J. Pathol. 2011, 178, 1221. 21356373 10.1016/j.ajpath.2010.11.076 PMC3070581

[18] Y. Zhu , J. Chen , C. Chen , R. Tang , J. Xu , S. Shi , X. Yu , Biomarker Res. 2025, 13, 11. 10.1186/s40364-025-00727-9 PMC11755887 39849659

[19] R. K. Jain , J. D. Martin , T. Stylianopoulos , Annu. Rev. Biomed. Eng. 2014, 16, 321. 25014786 10.1146/annurev-bioeng-071813-105259 PMC4109025

[20] M. Plodinec , M. Loparic , C. A. Monnier , E. C. Obermann , R. Zanetti‐Dallenbach , P. Oertle , J. T. Hyotyla , U. Aebi , M. Bentires‐Alj , R. Y. H. Lim , C.‐A. Schoenenberger , Nat. Nanotechnol. 2012, 7, 757. 23085644 10.1038/nnano.2012.167

[21] R. M. S. Sigrist , J. Liau , A. E. Kaffas , M. C. Chammas , J. K. Willmann , Theranostics 2017, 7, 1303. 28435467 10.7150/thno.18650 PMC5399595

[22] N. Subrahmanyam , H. Ghandehari , J. Pers. Med. 2021, 11, 88. 33572559 10.3390/jpm11020088 PMC7911184

[23] F. M. White , R. A. Gatenby , C. Fischbach , Cancer Res. 2019, 79, 2107. 31018939 10.1158/0008-5472.CAN-18-3937 PMC6497564

[24] I. Acerbi , L. Cassereau , I. Dean , Q. Shi , A. Au , C. Park , Y. Y. Chen , J. Liphardt , E. S. Hwang , V. M. Weaver , Integr. Biol. 2015, 7, 1120. 10.1039/c5ib00040h PMC4593730 25959051

[25] S. E. Reid , E. J. Kay , L. J. Neilson , A.‐T. Henze , J. Serneels , E. J. McGhee , S. Dhayade , C. Nixon , J. B. Mackey , A. Santi , K. Swaminathan , D. Athineos , V. Papalazarou , F. Patella , Á. Román‐Fernández , Y. ElMaghloob , J. R. Hernandez‐Fernaud , R. H. Adams , S. Ismail , D. M. Bryant , M. Salmeron‐Sanchez , L. M. Machesky , L. M. Carlin , K. Blyth , M. Mazzone , S. Zanivan , EMBO J. 2017, 36, 2373. 28694244 10.15252/embj.201694912 PMC5556271

[26] C. R. Pfeifer , C. M. Alvey , J. Irianto , D. E. Discher , Curr. Opin. Syst. Biol. 2017, 2, 103. 29082336 10.1016/j.coisb.2017.04.005 PMC5654613

[27] P. P. Provenzano , K. W. Eliceiri , J. M. Campbell , D. R. Inman , J. G. White , P. J. Keely , BMC Med. 2006, 4, 38. 17190588 10.1186/1741-7015-4-38 PMC1781458

[28] S. Kaushik , M. W. Pickup , V. M. Weaver , Cancer Metastasis Rev. 2016, 35, 655. 27914000 10.1007/s10555-016-9650-0 PMC5215979

[29] M. W. Conklin , R. E. Gangnon , B. L. Sprague , L. Van Gemert , J. M. Hampton , K. W. Eliceiri , J. S. Bredfeldt , Y. Liu , N. Surachaicharn , P. A. Newcomb , A. Friedl , P. J. Keely , A. Trentham‐Dietz , Cancer Epidemiol. Biomarkers Prev. 2018, 27, 138. 29141852 10.1158/1055-9965.EPI-17-0720 PMC5809285

[30] B. L. Sprague , P. M. Vacek , S. E. Mulrow , M. F. Evans , A. Trentham‐Dietz , S. D. Herschorn , T. A. James , N. Surachaicharn , A. Keikhosravi , K. W. Eliceiri , D. L. Weaver , M. W. Conklin , Cancer Epidemiol. Biomarkers Prev. 2021, 30, 80. 33082201 10.1158/1055-9965.EPI-20-0889 PMC7855820

[31] E. Klotzsch , M. L. Smith , K. E. Kubow , S. Muntwyler , W. C. Little , F. Beyeler , D. Gourdon , B. J. Nelson , V. Vogel , Proc. Natl. Acad. Sci. U. S. A. 2009, 106, 18267. 19826086 10.1073/pnas.0907518106 PMC2761242

[32] V. Vogel , Annu. Rev. Physiol. 2018, 80, 353. 29433414 10.1146/annurev-physiol-021317-121312

[33] L. Cao , J. Nicosia , J. Larouche , Y. Zhang , H. Bachman , A. C. Brown , L. Holmgren , T. H. Barker , ACS Nano 2017, 11, 7110. 28699736 10.1021/acsnano.7b02755 PMC5842356

[34] M. Chabria , S. Hertig , M. L. Smith , V. Vogel , Nat. Commun. 2010, 1, 135. 21139580 10.1038/ncomms1135 PMC3105298

[35] K. Selcuk , A. Leitner , L. Braun , F. L. Blanc , P. Pacak , S. Pot , V. Vogel , Matrix Biol. 2024, 125, 113. 38135164 10.1016/j.matbio.2023.12.006

[36] K. E. Kubow , R. Vukmirovic , L. Zhe , E. Klotzsch , M. L. Smith , D. Gourdon , S. Luna , V. Vogel , Nat. Commun. 2015, 6, 8026. 26272817 10.1038/ncomms9026 PMC4539566

[37] G. Efthymiou , A. Saint , M. Ruff , Z. Rekad , D. Ciais , E. Van Obberghen‐Schilling , Front. Oncol. 2020, 10, 641. 32426283 10.3389/fonc.2020.00641 PMC7203475

[38] Y. K. Bae , A. Kim , M. K. Kim , J. E. Choi , S. H. Kang , S. J. Lee , Hum. Pathol. 2013, 44, 2028. 23684510 10.1016/j.humpath.2013.03.006

[39] J. A. Eble , S. Niland , Clin. Exp. Metastasis 2019, 36, 171. 30972526 10.1007/s10585-019-09966-1

[40] D. Ortiz Franyuti , M. Mitsi , V. Vogel , Nano Lett. 2018, 18, 15. 28845674 10.1021/acs.nanolett.7b01617

[41] A. Krammer , H. Lu , B. Isralewitz , K. Schulten , V. Vogel , Proc. Natl. Acad. Sci. U. S. A. 1999, 96, 1351. 9990027 10.1073/pnas.96.4.1351 PMC15466

[42] A. Ariel , R. Hershkoviz , L. Cahalon , D. E. Williams , S. K. Akiyama , K. M. Yamada , C. Chen , R. Alon , T. Lapidot , O. Lider , Eur. J. Immunol. 1997, 27, 2562. 9368611 10.1002/eji.1830271015

[43] R. Tempest , S. Guarnerio , R. Maani , J. Cooper , N. Peake , Cancers 2021, 13, 2788. 34205140 10.3390/cancers13112788 PMC8199963

[44] C. Wu , S. M. Weis , D. A. Cheresh , J. Cell Sci. 2023, 136, jcs261483. 37870164 10.1242/jcs.261483 PMC10652044

[45] P. P. Provenzano , D. R. Inman , K. W. Eliceiri , P. J. Keely , Oncogene 2009, 28, 4326. 19826415 10.1038/onc.2009.299 PMC2795025

[46] M. W. Pickup , J. K. Mouw , V. M. Weaver , EMBO Rep. 2014, 15, 1243. 25381661 10.15252/embr.201439246 PMC4264927

[47] J. Martinez , P. C. Smith , Cells 2021, 10, 1046.33946660

[48] L. A. Tomko , R. C. Hill , A. Barrett , J. M. Szulczewski , M. W. Conklin , K. W. Eliceiri , P. J. Keely , K. C. Hansen , S. M. Ponik , Sci. Rep. 2018, 8, 12941. 30154546 10.1038/s41598-018-31126-w PMC6113240

[49] M. Egeblad , M. G. Rasch , V. M. Weaver , Curr. Opin. Cell Biol. 2010, 22, 697. 20822891 10.1016/j.ceb.2010.08.015 PMC2948601

[50] T. R. Cox , J. T. Erler , Dis. Models Mech. 2011, 4, 165. 10.1242/dmm.004077 PMC3046088 21324931

[51] S. Arnoldini , A. Moscaroli , M. Chabria , M. Hilbert , S. Hertig , R. Schibli , M. Béhé , V. Vogel , Nat. Commun. 2017, 8, 1793. 29176724 10.1038/s41467-017-01846-0 PMC5702617

[52] S. Hertig , M. Chabria , V. Vogel , Nano Lett. 2012, 12, 5162. 22938173 10.1021/nl302153h

[53] G. Baneyx , L. Baugh , V. Vogel , Proc. Natl. Acad. Sci. U. S. A. 2002, 99, 5139. 11959962 10.1073/pnas.072650799 PMC122735

[54] M. L. Smith , D. Gourdon , W. C. Little , K. E. Kubow , R. A. Eguiluz , S. Luna‐Morris , V. Vogel , PLoS Biol. 2007, 5, 268. 10.1371/journal.pbio.0050268 PMC1994993 17914904

[55] O. Peleg , T. Savin , G. V. Kolmakov , I. G. Salib , A. C. Balazs , M. Kröger , V. Vogel , Biophys. J. 2012, 103, 1909. 23199919 10.1016/j.bpj.2012.09.028 PMC3491717

[56] C. M. Fonta , S. Arnoldini , D. Jaramillo , A. Moscaroli , A. Oxenius , M. Behe , V. Vogel , Matrix Biol. Plus 2020, 8, 100046. 33543039 10.1016/j.mbplus.2020.100046 PMC7852196

[57] C. M. Fonta , T. Loustau , C. Li , S. Poilil Surendran , U. Hansen , D. Murdamoothoo , M. C. Benn , I. Velazquez‐Quesada , R. Carapito , G. Orend , V. Vogel , Matrix Biol. 2023, 116, 1. 36669744 10.1016/j.matbio.2023.01.002

[58] T. Loustau , C. Abou‐Faycal , W. Erne , P. A. zur Wiesch , A. Ksouri , T. Imhof , M. Mörgelin , C. Li , M. Mathieu , N. Salomé , G. Crémel , S. Dhaouadi , B. Bouhaouala‐Zahar , M. Koch , G. Orend , Matrix Biol. 2022, 108, 20. 35227929 10.1016/j.matbio.2022.02.007

[59] C. Spenlé , T. Loustau , H. Burckel , G. Riegel , C. Abou Faycal , C. Li , A. Yilmaz , L. Petti , F. Steinbach , C. Ahowesso , C. Jost , N. Paul , R. Carapito , G. Noël , F. Anjuère , N. Salomé , G. Orend , Front. Immunol. 2021, 12, 636108. 34290694 10.3389/fimmu.2021.636108 PMC8287883

[60] J. D. Brierley , M. Gospodarowicz , C. Wittekind , TNM classification of malignant tumours –8th Edition 2017.

[61] M. Kasprzycka , C. Hammarström , G. Haraldsen , Cell Adhes. Migr. 2015, 9, 83. 10.4161/19336918.2014.994901 PMC4594616 25793575

[62] R. T. Chiquet‐Ehrismann , Int. J. Biochem. Cell Biol. 2004, 36, 986. 15094113 10.1016/j.biocel.2003.12.002

[63] A. Cakir , I. Isik Gonul , O. Ekinci , B. Cetin , M. Benekli , O. Uluoglu , Pathol. Res. Pract. 2017, 213, 227. 28215639 10.1016/j.prp.2016.12.010

[64] M.‐M. Shao , S. K. Chan , A. M. C. Yu , C. C. F. Lam , J. Y. S. Tsang , P. C. W. Lui , B. K. B. Law , P.‐H. Tan , G. M. Tse , Virchows Arch. 2012, 461, 313. 22851038 10.1007/s00428-012-1289-9

[65] N. Rajković , X. Li , K. N. Plataniotis , K. Kanjer , M. Radulovic , N. T. Milošević , Front. Oncol. 2018, 8, 00348. 10.3389/fonc.2018.00348 PMC6125390 30214894

[66] D. Hanahan , R. A. Weinberg , Cell 2000, 100, 57. 10647931 10.1016/s0092-8674(00)81683-9

[67] R. Yerushalmi , R. Woods , P. M. Ravdin , M. M. Hayes , K. A. Gelmon , Lancet Oncol. 2010, 11, 174. 20152769 10.1016/S1470-2045(09)70262-1

[68] M. Pinamonti , F. Zanconati , Breast Cytopathology: Assessing the Value of FNAC in the Diagnosis of Breast Lesions, S Karger Ag, Switzerland 2017, Vol. 24.

[69] P. Bankhead , M. B. Loughrey , J. A. Fernández , Y. Dombrowski , D. G. McArt , P. D. Dunne , S. McQuaid , R. T. Gray , L. J. Murray , H. G. Coleman , J. A. James , M. Salto‐Tellez , P. W. Hamilton , Sci. Rep. 2017, 7, 16878. 29203879 10.1038/s41598-017-17204-5 PMC5715110

[70] T. Alkasalias , L. Moyano‐Galceran , M. Arsenian‐Henriksson , K. Lehti , Int. J. Mol. Sci. 2018, 19, 1532. 29883428 10.3390/ijms19051532 PMC5983719

[71] G. Gabbiani , O. Kocher , W. S. Bloom , J. Vandekerckhove , K. Weber , J. Clin. Invest. 1984, 73, 148. 6690475 10.1172/JCI111185 PMC424985

[72] P. Gugliotta , A. Sapino , L. Macrí , O. Skalli , G. Gabbiani , G. Bussolati , J. Histochem. Cytochem. 1988, 36, 659. 3367051 10.1177/36.6.3367051

[73] A. V. Ivshina , J. George , O. Senko , B. Mow , T. C. Putti , J. Smeds , T. Lindahl , Y. Pawitan , P. Hall , H. Nordgren , J. E. L. Wong , E. T. Liu , J. Bergh , V. A. Kuznetsov , L. D. Miller , Cancer Res. 2006, 66, 10292. 17079448 10.1158/0008-5472.CAN-05-4414

[74] C. w. Elston , I. o. Ellis , Histopathology 1991, 19, 403. 1757079 10.1111/j.1365-2559.1991.tb00229.x

[75] C. Spenlé , T. Loustau , D. Murdamoothoo , W. Erne , S. Beghelli‐de la Forest Divonne , R. Veber , L. Petti , P. Bourdely , M. Mörgelin , E.‐M. Brauchle , G. Cremel , V. Randrianarisoa , A. Camara , S. Rekima , S. Schaub , K. Nouhen , T. Imhof , U. Hansen , N. Paul , R. Carapito , N. Pythoud , A. Hirschler , C. Carapito , H. Dumortier , C. G. Mueller , M. Koch , K. Schenke‐Layland , S. Kon , A. Sudaka , F. Anjuère , Cancer Immunol. Res. 2020, 8, 1122. 32665262 10.1158/2326-6066.CIR-20-0074

[76] L. Bejarano , M. J. C. Jordāo , J. A. Joyce , Cancer Discovery 2021, 11, 933. 33811125 10.1158/2159-8290.CD-20-1808

[77] T. R. Cox , Nat. Rev. Cancer 2021, 21, 217. 33589810 10.1038/s41568-020-00329-7

[78] Z. Werb , P. Lu , Cancer J. 2015, 21, 250. 26222075 10.1097/PPO.0000000000000127 PMC4963227

[79] R. Kalluri , Nat. Rev. Cancer 2016, 16, 582. 27550820 10.1038/nrc.2016.73

[80] V. S. LeBleu , G. Taduri , J. O'Connell , Y. Teng , V. G. Cooke , C. Woda , H. Sugimoto , R. Kalluri , Nat. Med. 2013, 19, 1047. 23817022 10.1038/nm.3218 PMC4067127

[81] M. S. Samuel , J. I. Lopez , E. J. McGhee , D. R. Croft , D. Strachan , P. Timpson , J. Munro , E. Schröder , J. Zhou , V. G. Brunton , N. Barker , H. Clevers , O. J. Sansom , K. I. Anderson , V. M. Weaver , M. F. Olson , Cancer Cell 2011, 19, 776. 21665151 10.1016/j.ccr.2011.05.008 PMC3115541

[82] K. Wang , R. C. Andresen Eguiluz , F. Wu , B. R. Seo , C. Fischbach , D. Gourdon , Biomaterials 2015, 54, 63. 25907040 10.1016/j.biomaterials.2015.03.019 PMC4659482

[83] J. Najera , M. R. Rosenberger , M. Datta , Cancers 2023, 15, 3285. 37444394 10.3390/cancers15133285 PMC10340281

[84] N. F. Boyd , Q. Li , O. Melnichouk , E. Huszti , L. J. Martin , A. Gunasekara , G. Mawdsley , M. J. Yaffe , S. Minkin , PLoS One 2014, 9, 100937. 10.1371/journal.pone.0100937 PMC4091939 25010427

[85] A. Nicolas‐Boluda , J. Vaquero , L. Vimeux , T. Guilbert , S. Barrin , C. Kantari‐Mimoun , M. Ponzo , G. Renault , P. Deptula , K. Pogoda , R. Bucki , I. Cascone , J. Courty , L. Fouassier , F. Gazeau , E. Donnadieu , Elife 2021, 10, 58688. 10.7554/eLife.58688 PMC8203293 34106045

[86] M. Tamaoki , K. Imanaka‐Yoshida , K. Yokoyama , T. Nishioka , H. Inada , M. Hiroe , T. Sakakura , T. Yoshida , Am. J. Pathol. 2005, 167, 71. 15972953 10.1016/S0002-9440(10)62954-9 PMC1603439

[87] N. Jariwala , M. Ozols , M. Bell , E. Bradley , A. Gilmore , L. Debelle , M. J. Sherratt , Adv. Drug Delivery Rev. 2022, 185, 114240. 10.1016/j.addr.2022.114240 35378216

[88] A. Krammer , D. Craig , W. E. Thomas , K. Schulten , V. Vogel , Matrix Biol. 2002, 21, 139. 11852230 10.1016/s0945-053x(01)00197-4

[89] P. Moreno‐Layseca , J. Icha , H. Hamidi , J. Ivaska , Nat. Cell Biol. 2019, 21, 122. 30602723 10.1038/s41556-018-0223-z PMC6597357

[90] H. Hamidi , J. Ivaska , Nat. Rev. Cancer 2018, 18, 533. 30002479 10.1038/s41568-018-0038-z PMC6629548

[91] G. Jacquemet , D. M. Green , R. E. Bridgewater , A. von Kriegsheim , M. J. Humphries , J. C. Norman , P. T. Caswell , J. Cell Biol. 2013, 202, 917. 24019536 10.1083/jcb.201302041 PMC3776348

[92] R. O. Hynes , Science 2009, 326, 1216. 19965464 10.1126/science.1176009 PMC3536535

[93] K. Saini , S. Cho , L. J. Dooling , D. E. Discher , Matrix Biol. 2020, 85, 34. 31201857 10.1016/j.matbio.2019.06.001 PMC6906264

[94] Y. Zhao , K. Wei , H. Chi , Z. Xia , X. Li , Front. Immunol. 2022, 13, 1022808. 36389666 10.3389/fimmu.2022.1022808 PMC9650235

[95] S. C. Schwager , K. M. Young , L. A. Hapach , C. M. Carlson , J. A. Mosier , T. J. McArdle , W. Wang , C. Schunk , A. L. Jayathilake , M. E. Bates , F. Bordeleau , M. A. Antonyak , R. A. Cerione , C. A. Reinhart‐King , Elife 2022, 11, 74433. 10.7554/eLife.74433 PMC9767463 36475545

[96] A. Morello , J. Durand , M. Seite , I. Girault , G. Teppaz , V. Thepenier , C. Batty , A. Desselle , E. Wilhelm , M. Malloci , C. Mary , N. Poirier , J Immunother Cancer 2023, 11, 1370.

[97] K. Wang , B. R. Seo , C. Fischbach , D. Gourdon , Cel. Mol. Bioeng. 2016, 9, 1. 10.1007/s12195-015-0417-4 PMC4746220 26900407

[98] A. F. Frangi , W. J. Niessen , K. L. Vincken , M. A. Viergever , in Medical Image Computing and Computer‐Assisted Intervention — MICCAI’98, (Eds.: W. M. Wells , A. Colchester , S. Delp ), Springer, Cham 1998, pp. 130–137.

[99] Z. Püspöki , M. Storath , D. Sage , M. Unser , Adv. Anat. Embryol. Cell Biol. 2016, 219, 69. 27207363 10.1007/978-3-319-28549-8_3

