# Supplementary material for: Fibronectin Fibers Progressively Lose Their Tension in Invasive Human Breast Carcinoma while Being Tensed in DCIS and Healthy Breast Tissue
Source: Adv Sci (Weinh). 2025 Jun 5;12(31):e04351. doi: 10.1002/advs.202404351 (PMC12376670; doi:10.1002/advs.202404351)
Supplement: Supplementary file 1 — Supporting Information [file ADVS-12-e04351-s001.docx]

Supporting Information:

**Fibronectin Fibers Progressively Lose Their Tension in Invasive Human Breast Carcinoma, While Being Tensed in DCIS and Healthy Breast Tissue**

Arnaud Miéville^1*^, Charlotte M. Fonta^1*^, Cornelia Leo^2^, Lucine Christe^3^, Jörg Goldhahn^4^, Gad Singer^5‡^, Viola Vogel^1‡^

**Table S1:** Clinical classification of patients

**Figure S1:** Representative confocal images from each patient stained for Fibronectin, TNC, untensed Fibronectin fibers, and collagen fibers.

**Figure S2:** Fibronectin pixel ratio.

**Figure S3:** SHG pixel ratio.

**Figure S4**: Spatial proximity analysis between cell and untensed Fibronectin fibers

**Figure S5:** Representative confocal images from each patient stained for cancer cell markers and untensed Fibronectin fibers.

**Figure S6:** Representative confocal images of tumor cells for grade 2 and 3 invasive tumor pT2.

**Figure S7:** Ratiometric analysis of α_5_ versus α_v_ integrin.

***Table S1: Clinical classification of patients.*** The "p" stands for "pathologic stage", also called surgical stage, assessment of the tumor from a tumor biopsy^48^. The "T" followed by a number between 0 and 4 refers to the size of the tumor, with increasing values associated with larger tumor sizes. T1 (a through c) refers to tumors up to 2 cm in size. Within the T1 category, tumors are further divided into subcategories based on size, with the letters a, b, and c indicating tumor sizes in the ranges [0.1 cm and 0.5 cm], [0.5 cm to 1 cm], and [1 cm to 2 cm], respectively. The size of tumors smaller than 0.1cm is referred to as "Tmi". T2 refers to tumors between 2cm and 5cm, and T3 refers to tumors larger than 5cm. Finally, Tis refers to carcinoma in situ (DCIS) or Paget's disease, the non-invasive precursor of breast carinoma. NST: No Special Type


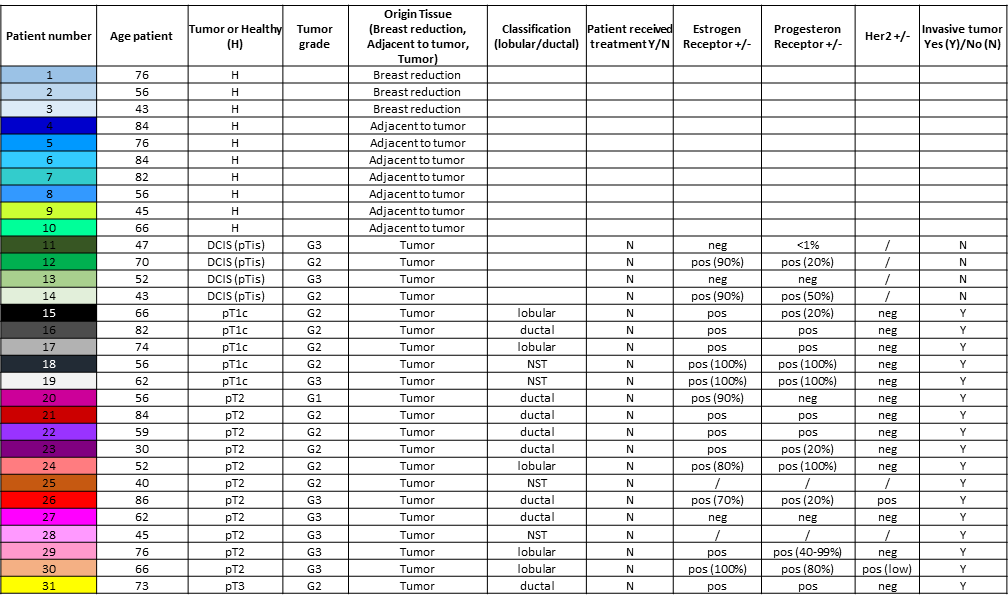


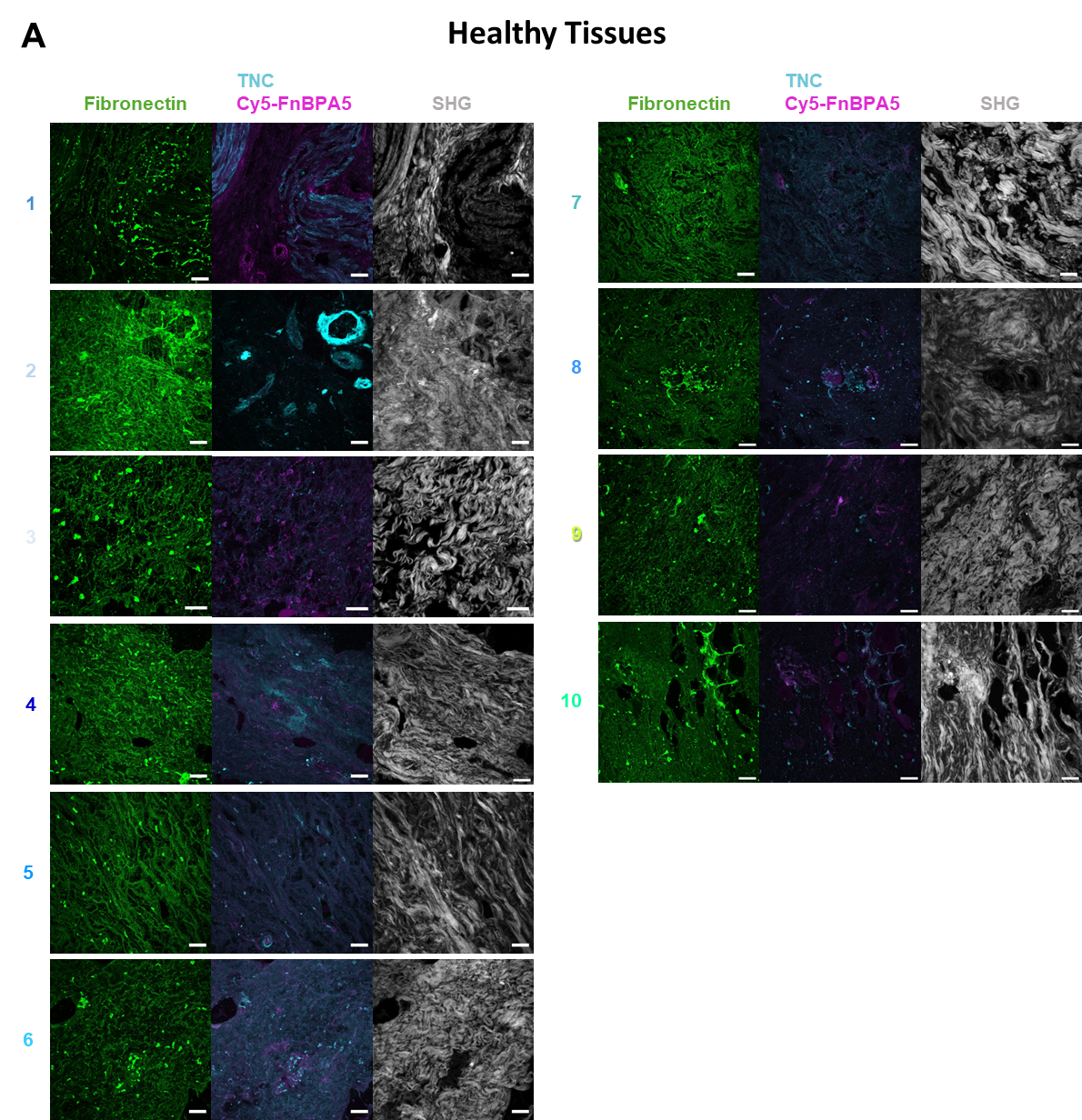


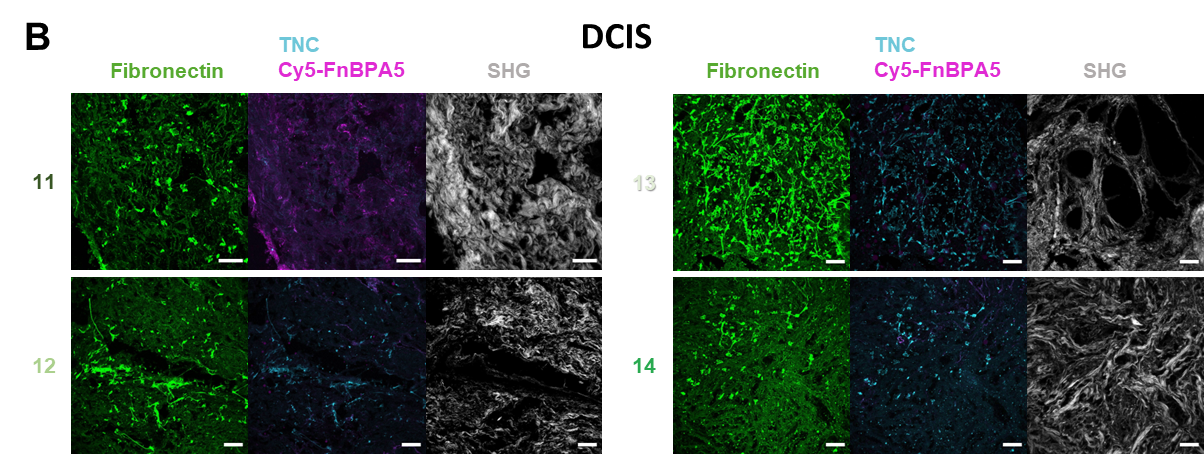


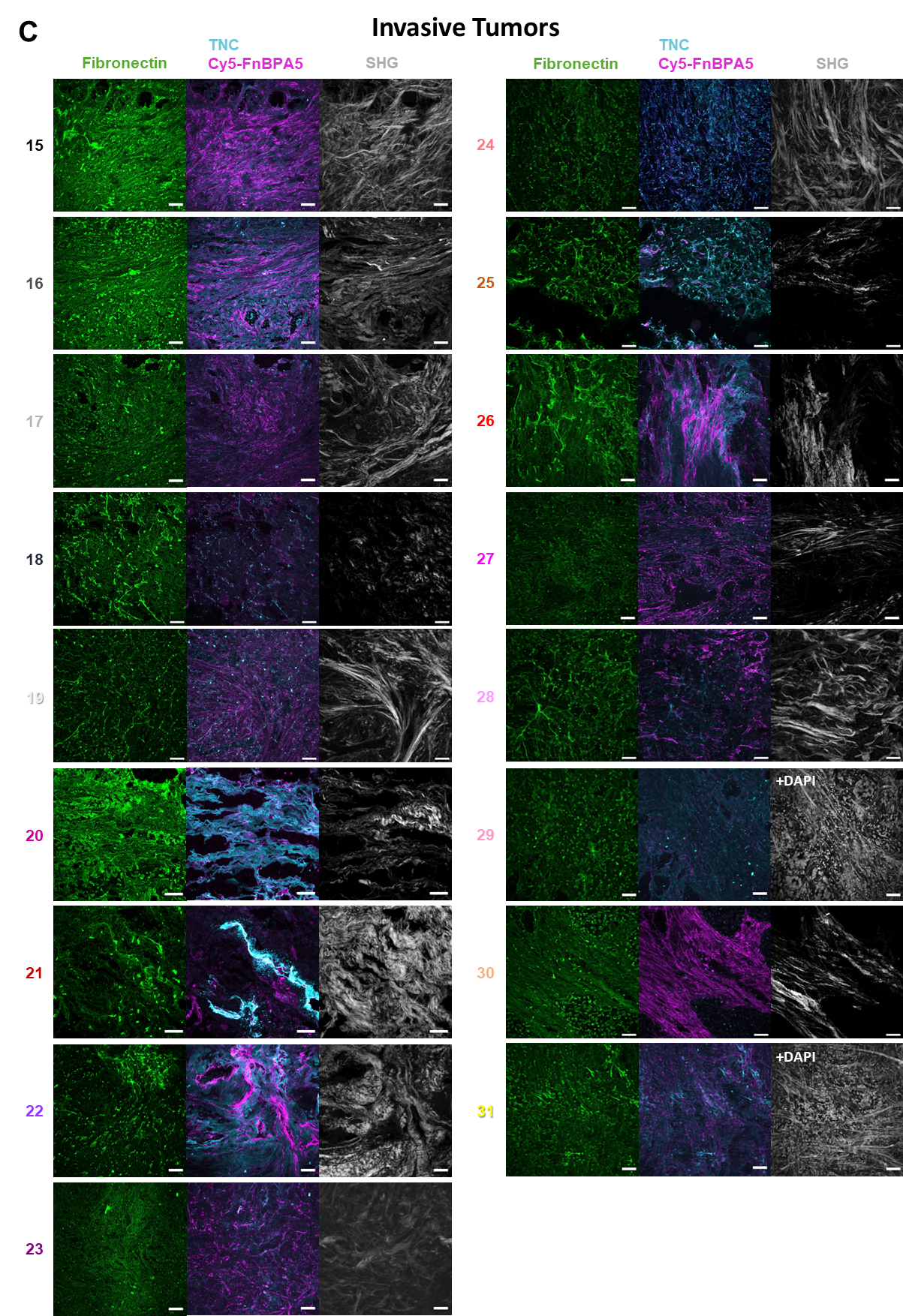


***Fig. S1:*** Representative confocal images of cryosections for each patient from Table S1 stained with a polyclonal Fibronectin antibody (green) to visualize the presence of all Fibronectin fibers, co-stained with Cy5-FnBPA5 tension probe (magenta) to visualize the locations of untensed Fibronectin fibers, as well as with a monoclonal TNC antibody (cyan), and SHG detection to localize dense bundles of collagen fibrils (gray) for the following tissues: Healthy breast tissue (**A**), DCIS (**B**), and Invasive Tumors (**C**). Scale bars: 50µm. Each number is color coded with the patient’s specific color (Table S1).


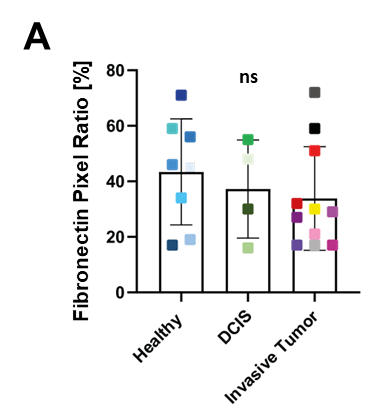


***Fig.S2: Fibronectin pixel ratio.* A:**Pixel ratio of the polyclonal Fibronectin was assessed as the percentage of positive pixels above a defined threshold for the polyclonal Fibronectin channel, normalized to the total number of pixels in the studied area. Multiple images were analyzed for each patient and the average of their total coverage was plotted. Each point represents one patient. Mean ± SD. Kruskal-Wallis test with Dunn’s multiple comparison test. The coloring of the data points correlates with the patient classifications as shown in Table S1.


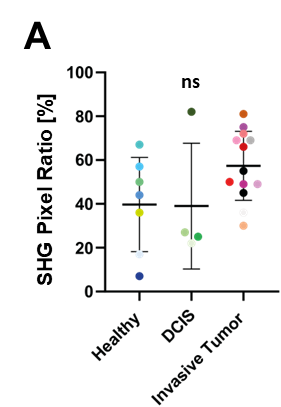


***Fig.S3: SHG pixel ratio.* A:**Pixel ratio of SHG was assessed as the percentage of positive pixels above a defined threshold for the SHG channel, normalized to the total number of pixels in the studied area. Multiple images were analyzed for each patient and the average of their total coverage was plotted. Each point represents one patient. Mean ± SD. Kruskal-Wallis test with Dunn’s multiple comparisons test. The coloring of the data points correlates with the patient classifications as shown in Table S1


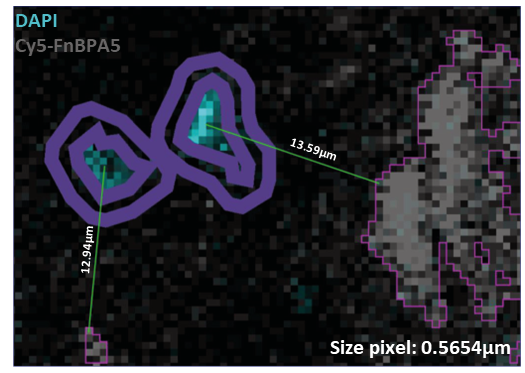


***Fig.S4: Spatial proximity analysis between cells and untensed Fibronectin fibers.*** Example of a proximity analysis performed by QuPath 0.4.2. Cells are stained with DAPI (cyan) and highlighted in purple with the plug-In “Cell detection”. Cy5-FnBPA5 signal is shown in gray scale. Masks containing positive pixels for Cy5-FnBPA5 above a certain threshold are depicted in magenta. The plug-In “Spatial analysis – Distance to annotations 2D”, allows to compute the smaller distance between the cell center and the closest positive pixel for Cy5-FnBPA5, here shown in green.

***
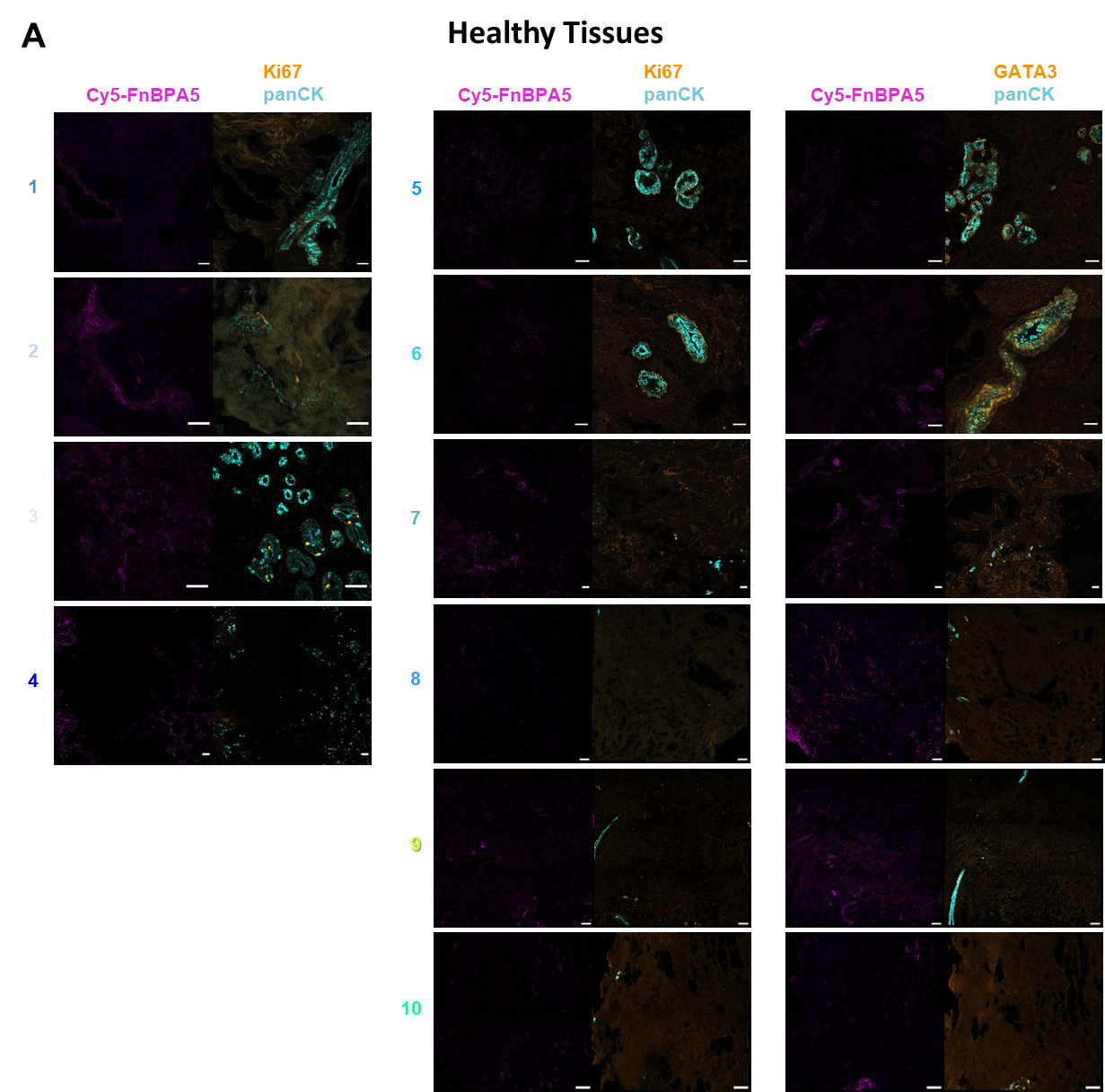
***

***
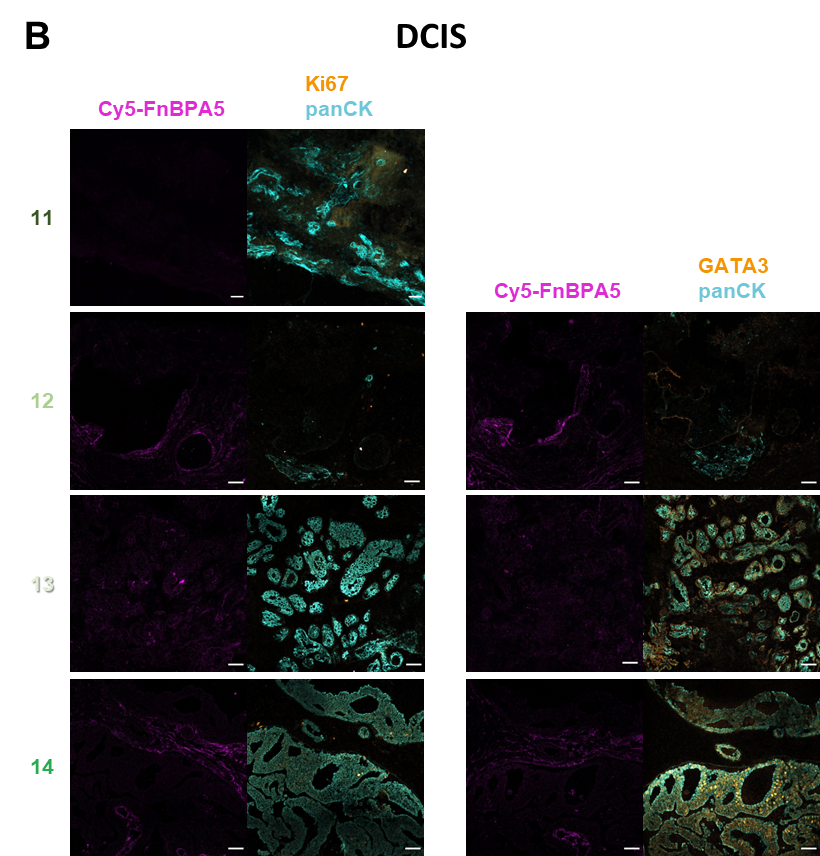
***

***
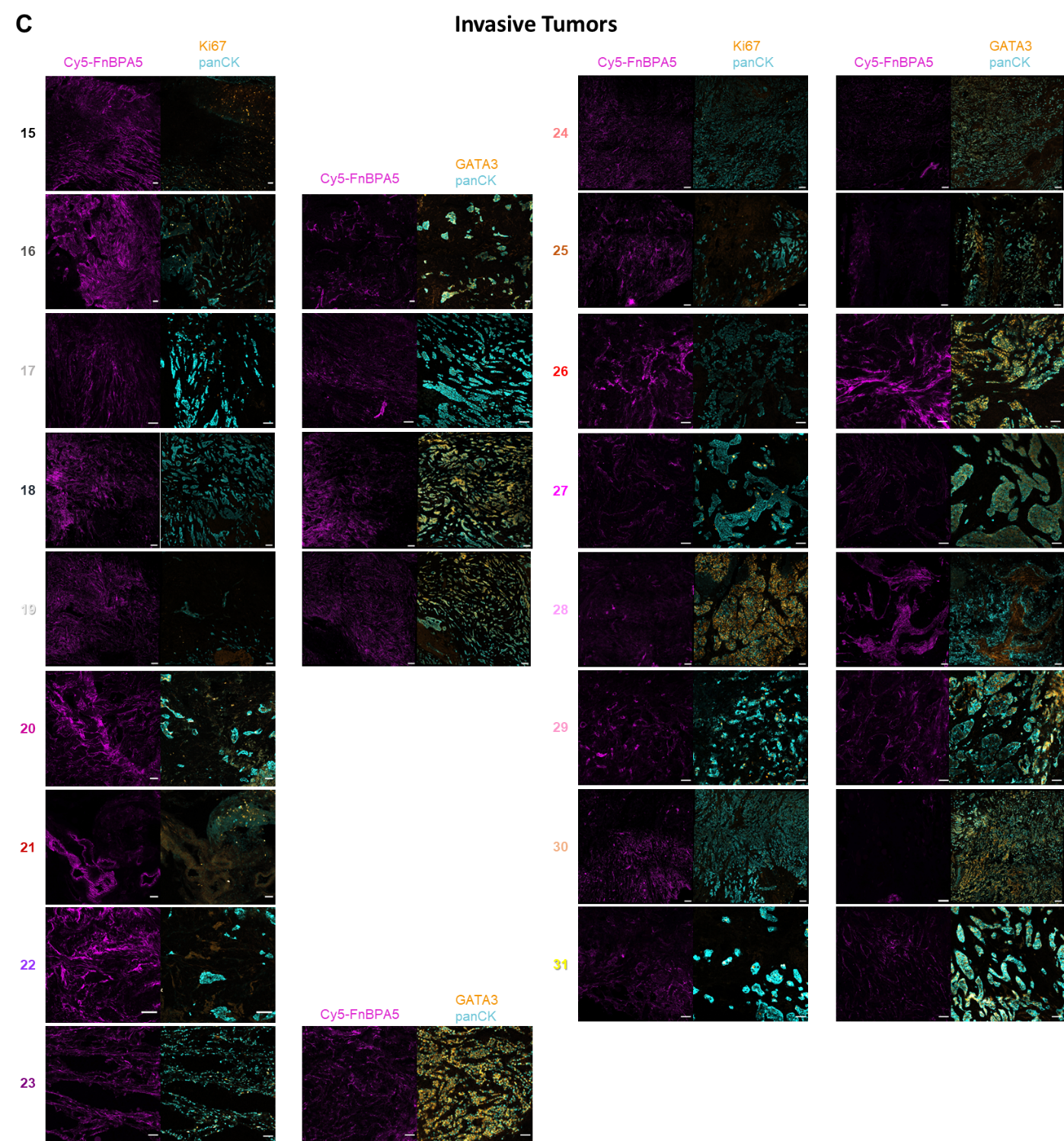
***

***Fig. S5:*** Representative confocal images of cryosections for each patient from Table S1 co-stained with either Ki67 (orange) and panCK (cyan), or GATA3 (orange) and panCK (cyan) to visualize carcinoma cells, together with the Cy5-FnBPA5 tension probe (magenta) to visualize the locations of untensed Fibronectin fibers for the following tissues: Healthy breast tissues (**A**), DCIS (**B**), and Invasive tumor (**C**). Scale bars: 100µm. Each number is color coded with the patient specific color (Table S1). Not all patients were stained with the GATA3-panCK antibody combination.


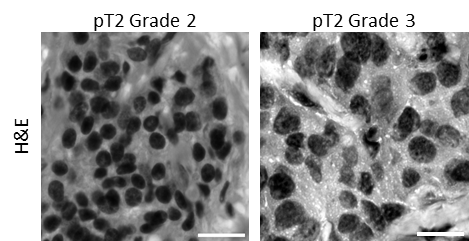


***Fig. S6:*** ***Representative H&E images of tumor cells from Invasive Tumor pT2 grade 2 and 3***. Scale bar: 20µm


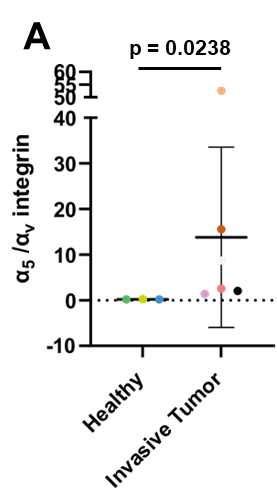


***Fig. S7:*** ***Ratiometric analysis of α_5_ versus α_v_ integrin.*** Data are expressed as the ratio of positive cells for α_5_ antibody above a certain threshold to positive cells for α_v_ antibody above a certain threshold. Multiple images were analyzed for each patient and the average ratio was plotted. Each point represents one patient. Mean ± SD. Mann-Whitney test. The coloring of the data points correlates with the patient classifications as shown in Table S1.
